# Associations between obesity and arterial stiffness assessed by cardio-ankle vascular index in healthy children and adolescents

**DOI:** 10.3389/fcvm.2025.1633849

**Published:** 2025-09-09

**Authors:** Yuko Horikoshi, Rie Sakuraba-Hirata, Nina Suzuki, Yuki Inomata, Moe Oikawa, Toa Kotani, Takumu Watanabe, Eri Takahashi, Kanako Okazaki, Masayuki Hoshi, Yasuhiro Endo, Tatsuya Nakanowatari, Hiroaki Abe, Yasuaki Kusumoto, Mieko Yokozuka, Yoshitaka Shiba, Yoshinobu Tanaka, Akihiko Asao, Shigeki Kurasawa, Yasuchika Takeishi, Akiomi Yoshihisa

**Affiliations:** ^1^ Department of Clinical Laboratory Sciences, Fukushima Medical University School of Health Sciences, Fukushima, Japan; ^2^ Department of Physical Therapy, Fukushima Medical University School of Health Sciences, Fukushima, Japan; ^3^ Department of Occupational Therapy, Fukushima Medical University School of Health Sciences, Fukushima, Japan; ^4^ Department of Cardiovascular Medicine, Fukushima Medical University School of Medicine, Fukushima, Japan

**Keywords:** cardio-ankle vascular index, Rohrer index, body fat percentage, percentage of overweight, body mass index, obesity paradox

## Abstract

**Aims:**

Cardio-ankle vascular index (CAVI) is a non-invasive method for evaluating arterial stiffness. In adults, CAVI has been reported to show negative correlation with body mass index (BMI) known as the “obesity paradox”; however, whether this also applies to children remains unclear. In addition, childhood obesity is a problem in developed countries, and the utility of CAVI in children has not yet been clarified. We here aimed to clarify the relationship between obesity parameters and CAVI in healthy children.

**Methods:**

This was a cross-sectional study conducted in 2024. We evaluated CAVI and its associated factors in 590 children aged 6–15 years (mean age: 10.5 years, 51.0% female). Additionally, obesity parameters, including the Rohrer index, percentage of overweight (POW), body fat percentage and body fat mass determined by bioelectrical impedance analysis, and BMI were assessed. The participants were categorized into groups based on the obesity parameters.

**Results:**

CAVI decreased as obesity level increased, showing the lowest CAVI in the highest obesity category. CAVI was positively correlated with age (*R* = 0.18, *p* < 0.05), and was negatively correlated with Rohrer index, body fat percentage, body fat mass, POW, BMI, and heart rate, (*R* = −0.33, *R* = −0.23, *R* = −0.14, *R* = −0.30, *R* = −0.19, *R* = −0.14, respectively; *p* < 0.01 for all). In contrast, CAVI showed no significant correlation with blood pressure or body weight. Furthermore, multiple linear regression analyses after adjusting for possible obesity-related factors including age, sex, blood pressure, and heart rate, showed that all obesity parameters were independent predictors of CAVI.

**Conclusion:**

Obesity parameters are negatively and independently associated with CAVI in healthy children.

## Introduction

Cardio-ankle vascular index (CAVI) is a non-invasive and simple method for evaluating arterial stiffness and arteriosclerosis, and has been reported to be useful for predicting cardiovascular disease in adults (1–5). However, there is a negative correlation between CAVI and body mass index (BMI) in healthy adults, known as the “obesity paradox” (6, 7). Namely, CAVI tends to be lower in obese adults, who are at increased risk for hypertension, diabetes, dyslipidemia, and cardiovascular disease. However, since there are possible obesity-related factors (age, sex, blood pressure, heart rate, body fat mass, vascular characteristics, and etc.), it is not clear enough about the cause of “obesity paradox”.

Childhood obesity is a significant problem in developed countries (8, 9). Pathological arteriosclerosis such as intimal thickening has been reported to progress with age, starting from the first year of life (10). Since childhood obesity is likely to continue into adulthood, with associated increases in morbidity and mortality, early intervention is crucial. Evaluating arterial stiffness from childhood is important for preventing arteriosclerosis and reducing the risk of future cardiovascular diseases (11). However, there are only a few studies on CAVI and BMI in children (12, 13), and its validity and accuracy in these populations remain unclear. Additionally, BMI is not necessarily appropriate for assessing childhood obesity. BMI is calculated based on height and weight, varies greatly during growth for specific ages, and thus fixed cut-off values for obesity cannot be set for children (14, 15). Instead, Rohrer index is known to be appropriate for assessing childhood obesity (16, 17). In addition, percentage of overweight (POW) has reflects the actual condition related to the physical status of children with regard to fatness in puberty to a greater extent than BMI (14). Furthermore, the bioelectrical impedance analysis (BIA) method can classify childhood obesity in more accurate than BMI (18, 19).

Furthermore, CAVI measures the combined properties of the aorta, femoral artery, and tibial artery. The aorta is an elastic artery, whereas the femoral and tibial arteries are muscular vessels (20). The difference in the ratio of elastic and muscular blood vessels in the total length of the measured vessels may affect CAVI. To clarify impact of property of elastic and muscular blood vessels on CAVI, we newly tried to use muscular vessel ratio (MVR) in the study.

Therefore, the purpose of the present study was (A) to evaluate the validity and accuracy of CAVI measurement, (B) to clarify the relationships between CAVI and several obesity parameters (Rohrer index, POW, BMI, body fat percentage and body fat mass determined by BIA), and (C) to examine the factor which influence “obesity paradox” such as blood pressure, MVR etc., in healthy children and adolescents.

## Methods

### Study population

**Figure 1:**
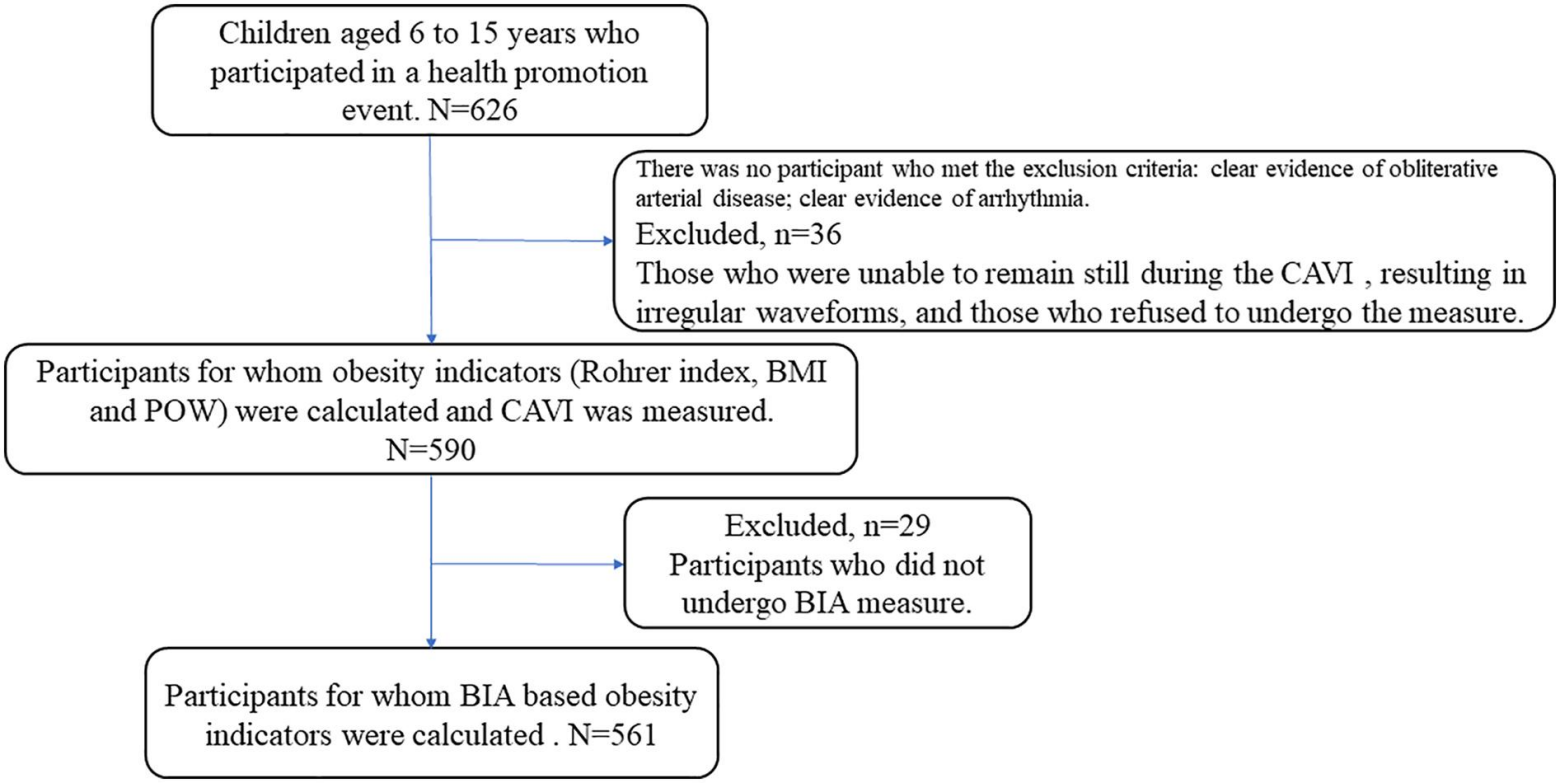
Study flow chart. This study included 626 children and adolescents aged 6–15 years. Of the 626 study participants, 36 were excluded because they were unable to maintain a resting state during CAVI measurement or showed irregular waveforms. Furthermore, 29 participants did not undergo BIA measurement. The analysis was ultimately conducted on 561 participants.

Study flow chart is presented in Figure 1. This was a cross-sectional study of 626 children and adolescents aged 6–15 years (mean 10.5 years; 51% females) who took part in a health promotion event held by Fukushima Medical University School of Health Sciences in 2024. We measured their CAVI, height, weight, Rohrer index, POW, and BMI, as well as their body fat percentage and body fat mass, assessed using BIA. The exclusion criteria were clear evidence of obliterative arterial disease; clear evidence of arrhythmia such as atrial fibrillation; and refusal to undergo measurement. There were no subjects who met the exclusion criteria. However, of the 626 study participants, 36 were excluded due to inability to remain still or irregular waveforms, resulting in a final analysis of 590 individuals. Out of the 590 participants, 561 were measured body fat percentage and body fat mass determined by BIA.

To evaluate the accuracy and validity of CAVI measurements, we measured the length of the participants' blood vessels over their clothing with a tape measure and compared it to the vessel length calculated from height based on CAVI measurements. CAVI is consisted of the combined properties of elastic vessel (i.e., aorta) and muscular vessel (i.e., femoral and tibial arteries) (20). To clarify the impact of property of elastic and muscular blood vessels on CAVI, we tried to assess MVR. Based on previous reports (20–22), vascular length was defined as follows: the distance from the second intercostal space to the base of the foot (L1), the distance from the base of the foot to the knee (L2), and the distance from the knee to the ankle (L3). The MVR was defined as (L2 + L3)/(L1 + L2 + L3).

We examined the relationship between each obesity parameter (Rohrer index, body fat percentage, POW, and BMI) and CAVI. The Rohrer index is used as an indicator of childhood obesity and is calculated as follows: weight (kg)/height (m^3^) (16, 17). Body fat percentage was measured using BIA. According to the criteria for childhood obesity adopted by the Ministry of Education, Culture, Sports, Science and Technology in Japan, a child was considered to be obese when the POW exceeded 20%. POW = 20% means that one's body weight is 120% of standard body weight, which is defined as the mean body weight corresponding to the height for that age obtained from the national statistics for Japanese school children in 2000. BMI is calculated as follows: weight (kg)/height (m^2^) (16, 18, 23, 24). The study participants were divided into groups based on Rohrer index (lean, moderately lean, normal, moderately obese, and obese) (17, 25), body fat percentage (underweight, −normal, +normal, overweight, obese), POW (underweight, normal, mild obesity, moderate obesity, severe obesity), and BMI (underweight, normal weight, and overweight-obesity) (15, 26–28). We compared CAVI, height, weight, Rohrer index, body fat percentage, POW, and BMI, blood pressure, heart rate (HR), and MVR among the groups. The study protocol was approved by the Research Ethics Committee of Fukushima Medical University (No.2021-186, REC2023-029). The participants were given a written explanation of the study, and consent for participation was obtained from both the participants and their guardians.

### CAVI measurement

CAVI was measured automatically using VaSera VS-3000TE (Fukuda Denshi Co., Ltd., Tokyo, Japan) with the participant in the supine position (20). Cuffs were attached bilaterally to the upper arms and ankles. The cuff size for the upper arm was selected based on the measured circumference from the following three sizes: S (170–220 mm), M (220–300 mm), and L (300–390 mm). Electrocardiogram electrodes were placed on both wrists and a microphone was placed on the sternum. The average CAVI from both sides was used for analysis.

### Measurements of body fat percentage and body fat mass

Body fat percentage and body fat mass were measured using MC-780A-N (TANITA Co., Ltd., Tokyo, Japan) with the participant in the standing position. Body composition was estimated to be using the multi-frequency bioelectrical impedance analysis method by measuring electrical resistance, which reflects the ease of current flow through the body (23).

### Statistical analysis

Normality was confirmed using the Shapiro–Wilk test. Normally distributed data are expressed as mean ± standard deviation. Categorical variables are expressed as numbers (percentages), and the chi-square test was used for comparison. Comparison of parametric variables between groups was conducted using one-way analysis of variance, followed by Bonferroni *post-hoc* analysis. The associations between CAVI and obesity parameters (i.e., POW, Rohrer index, BMI, and body fat percentage) were examined using Pearson's correlation analysis for parametric variables. The difference between CAVI-based and manually measured vascular lengths was assessed with linear regression analysis and Bland-Altman analysis. Multiple linear regression analyses were performed to assess the associations between CAVI and obesity parameters, as well as variables commonly associated with CAVI (e.g., age, sex, and blood pressure, HR). A *p* value of <0.05 was considered statistically significant in all analyses. All analyses were conducted using IBM SPSS Statistics version 26 (IBM, Armonk, NY, USA).

## Results

**Figure 2:**
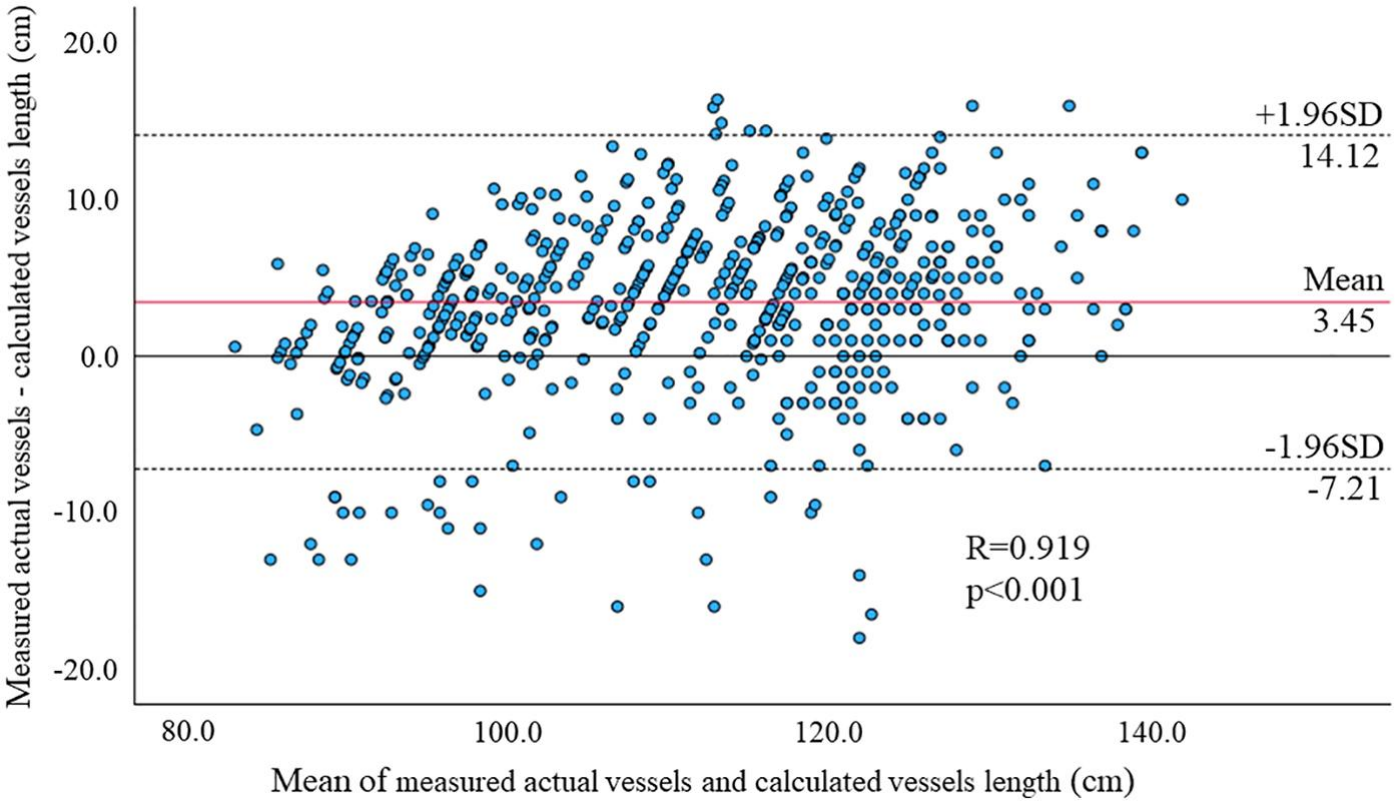
Bland-Altman plots of manually measured and CAVI-based vessel lengths for each parameter. The linear regression analysis revealed strong correlations between the vascular lengths measured by the two methods (*R* = 0.922, *p* < 0.001), and the Bland-Altman analysis showed mean differences were 3.45 ± 5.36. These results suggest excellent agreement between the two measurements.

We firstly examined the accuracy and validity of CAVI measurements in children. Linear regression analysis revealed strong correlations between the manually measured and CAVI-based vessel lengths (*R* = 0.922, *p* < 0.001, Figure 2), and the Bland-Altman analysis showed a mean difference of 3.44 ± 5.36, indicating excellent concordance.

**Figure 3:**
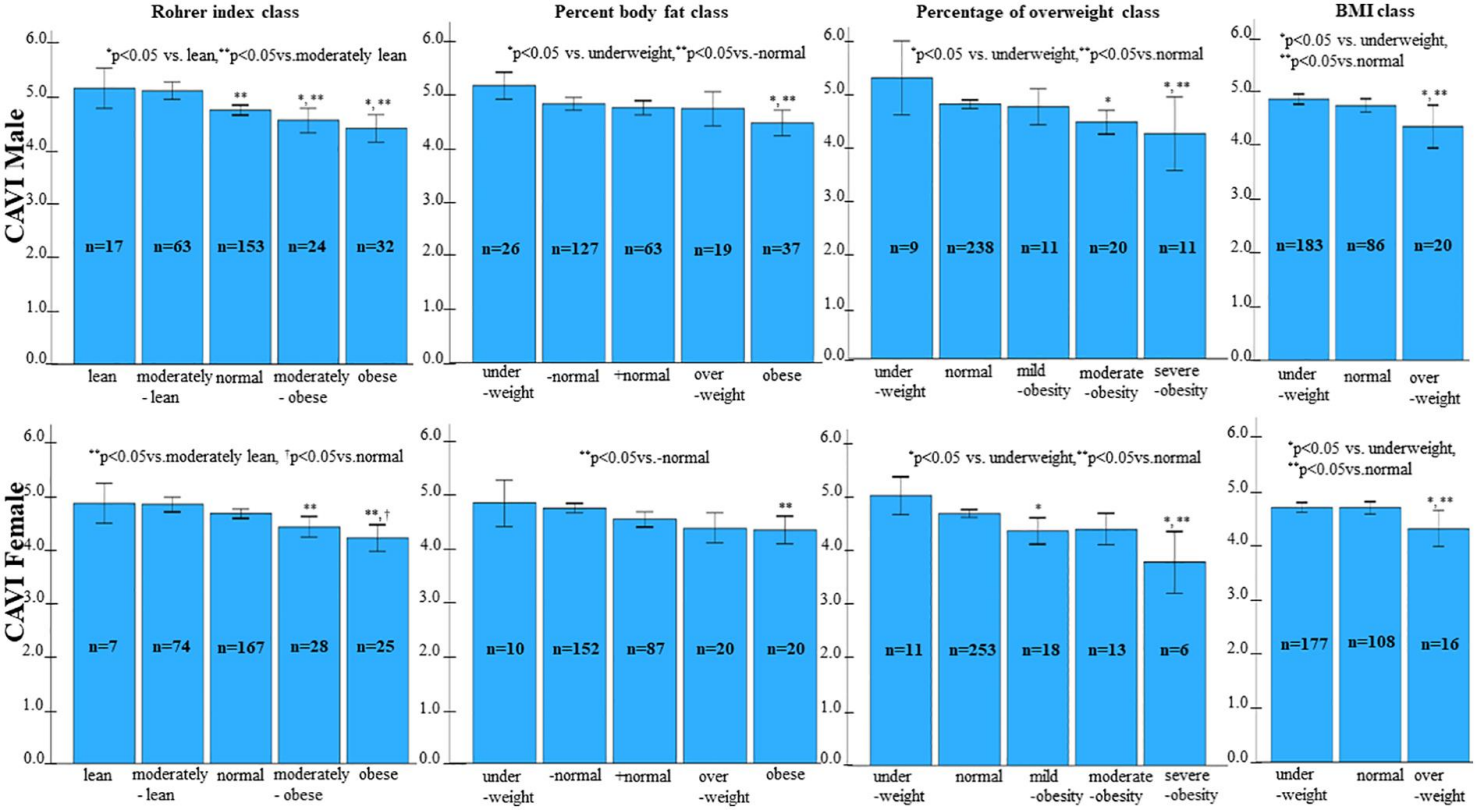
Comparison of CAVI by category of percentage of overweight. In both males and females, CAVI was lowest in the group with the highest obesity.

Next, we examined the relationship between each obesity parameter (Rohrer index, body fat percentage, POW, and BMI) and CAVI. The results of CAVI comparisons based on obesity classification using the Rohrer index are shown in Table 1. CAVI decreased as obesity levels increased, showing the lowest CAVI in the groups with the highest obesity (*p* < 0.001). Similarly, CAVI decreased as the degree of obesity increased for other obesity parameters (Table 2, body fat percentage based on BIA; Table 3, POW; and Table 4, BMI). Additionally, these highest obesity groups exhibited the highest SBP (*p* < 0.001; Tables 1–4). With respect to age and sex, except for the body fat percentage classification (Table 2), there were no differences in sex among the groups. Except for body fat percentage classification (Table 2), age showed significant differences without specific tendency. In both the male and female participants, CAVI was lowest in the group with the highest obesity (Figure 3). Comparisons of other parameters stratified by gender based on obesity classes are shown in Supplementary Tables (1a, Rohrer index-male; 1b, Rohrer index-female; 2a, body fat percentage based on BIA-male; 2b, body fat percentage based on BIA-female; 3a, POW-male; 3b, POW-female; 4a, BMI-male; 4b, BMI-female).

**Table 1:** Cardio-ankle vascular index of participants by Rohrer index classification (*n* = 590).

Rohrer index categories	Total	Lean	Moderately lean	Normal	Moderately obese	Obese	*p* value
Rohrer index (kg/m^3^)		Rohrer index < 100	100 ≤ Rohrer index < 115	115 ≤ Rohrer index < 145	145 ≤ Rohrer index < 160	160 ≤ Rohrer index	
Rohrer index (kg/m^3^)	129.1 ± 22.3	101.5 ± 13.5	108.6 ± 4.3	127.8 ± 8.4	149.7 ± 6.8	178.6 ± 20.9	<0.001
*n*	590	24	137	320	52	57	-
CAVI	4.7 ± 0.6	5.0 ± 0.6	4.9 ± 0.6	4.7 ± 0.6**	4.5 ± 0.5*^,^**	4.3 ± 0.7*^,^**^,^***	<0.001
Age, *n* (%)	10.5 ± 2.6	11.9 ± 1.9	11.1 ± 2.3	10.1 ± 2.8*^,^**	10.7 ± 2.4	10.8 ± 2.4	<0.001
Female, *n* (%)	301 (51.0)	7 (29.2)	74 (54.0)	167 (52.2)	28 (53.8)	25 (43.9)	0.160
SBP (mmHg)	116.9 ± 10.5	119.8 ± 10.0	116.0 ± 10.0	115.7 ± 10.4	119.2 ± 8.8	122.0 ± 11.9**^,^***	<0.001
DBP (mmHg)	68.7 ± 6.7	68.6 ± 6.7	68.9 ± 6.5	68.3 ± 6.7	69.5 ± 6.4	69.7 ± 7.1	0.564
MBP (mmHg)	84.8 ± 7.1	85.7 ± 7.2	84.6 ± 6.9	84.1 ± 7.2	86.0 ± 6.5	87.1 ± 7.7 ***	0.029
PP (mmHg)	48.2 ± 8.3	51.3 ± 6.9	47.1 ± 8.0	47.4 ± 8.1	49.8 ± 6.8	52.3 ± 9.9**^,^***	<0.001
HR (bpm)	78.9 ± 14.6	73.6 ± 10.8	77.3 ± 14.6	79.1 ± 14.5	80.6 ± 17.6	82.0 ± 12.5	0.101
MVR (%)	48.6 ± 0.4	48.9 ± 0.3	48.7 ± 0.4	48.5 ± 0.5*^,^**	48.6 ± 0.3	48.6 ± 0.4	<0.001
Height (cm)	142.5 ± 16.1	155.6 ± 13.8	145.8 ± 13.7*	139.5 ± 17.0*^,^**	144.0 ± 13.9*	145.0 ± 14.2	<0.001
Body weight (kg)	38.7 ± 14.4	38.6 ± 9.6	34.4 ± 9.3	36.2 ± 13.2	46.0 ± 13.2**^,^***	56.2 ± 18.4*^,^**^,^***^,^****	<0.001
POW (%)	2.6 ± 17.9	−16.3 ± 10.0	−12.9 ± 3.9	0.9 ± 7.4*^,^**	19.0 ± 6.6*^,^**^,^***	42.5 ± 17.2*^,^**^,^***^,^****	<0.001
BMI (kg/m^2^)	18.4 ± 3.8	15.7 ± 2.1	15.8 ± 1.4	17.8 ± 2.4*^,^**	21.6 ± 2.5*^,^**^,^***	25.9 ± 4.1*^,^**^,^***^,^****	<0.001
BIA *n* = 561	561	17	132	307	50	55	-
Body fat percentage (%)	20.0 ± 9.6	10.1 ± 4.2	13.4 ± 5.3	18.5 ± 6.3*^,^**	29.2 ± 5.3*^,^**^,^***	38.7 ± 6.8*^,^**^,^***^,^****	<0.001
Body fat mass (kg)	8.8 ± 7.3	3.8 ± 1.4	4.8 ± 2.6	7.3 ± 4.5**	14.2 ± 5.7*^,^**^,^***	22.8 ± 10.3*^,^**^,^***^,^****	<0.001
Lean body mass (kg)	30.3 ± 9.0	34.7 ± 8.9	29.8 ± 7.6	29.1 ± 9.4	32.7 ± 7.5	34.3 ± 9.1**^,^***	<0.001

CAVI, cardio-ankle vascular index; SBP, systolic blood pressure; DBP, diastolic blood pressure; MBP, mean blood pressure; PP, pulse pressure; HR, heart rate; MVR, muscular vessel ratio; POW, percentage of overweight; BMI, body mass index; BIA, bioelectrical impedance analysis.

* *p* < 0.05 vs. lean.

** *p* < 0.05 vs. moderately lean.

*** *p* < 0.05 vs. normal.

**** *p* < 0.05 vs. moderately obese.

**Table 2:** Cardio-ankle vascular index of participants by body fat percentage classification (*n* = 561).

Body fat percentage categories	Total	Underweight	−Normal	+Normal	Overweight	Obese	*p* value
Body fat percentage (%)	20.0 ± 9.6	7.4 ± 3.4	14.8 ± 4.6	22.9 ± 4.7	29.6 ± 3.5	39.2 ± 6.2	<0.001
*n*	561	36	279	150	39	57	-
CAVI	4.7 ± 0.6	5.1 ± 0.6	4.7 ± 0.6	4.6 ± 0.6*	4.5 ± 0.6*	4.4 ± 0.7*^,^**	<0.001
Age, *n* (%)	10.6 ± 2.6	10.1 ± 2.9	10.4 ± 2.7	10.7 ± 2.7	10.8 ± 2.1	11.1 ± 2.3	0.167
Female, *n* (%)	289 (51.5)	10 (27.8)	152 (54.5)	87 (58.0)	20 (51.3)	20 (35.1)	0.001
SBP (mmHg)	116.9 ± 10.5	111.5 ± 9.1	114.9 ± 10.1	118.6 ± 9.9*^,^**	120.9 ± 8.5*^,^**	122.7 ± 11.8*^,^**	<0.001
DBP (mmHg)	68.7 ± 6.7	64.9 ± 5.8	68.3 ± 6.9*	69.4 ± 6.4*	70.4 ± 5.7*	69.8 ± 7.0*	0.001
MBP (mmHg)	84.7 ± 7.2	80.5 ± 6.5	83.8 ± 7.2	85.8 ± 6.7*	87.2 ± 6.0*^,^**	87.4 ± 7.7*^,^**	<0.001
PP (mmHg)	48.2 ± 8.3	46.6 ± 6.2	46.6 ± 8.0	49.3 ± 8.2**	50.5 ± 6.6	52.9 ± 9.5*^,^**^,^***	<0.001
HR (bpm)	78.9 ± 14.8	75.6 ± 13.4	78.7 ± 14.7	78.0 ± 14.1	80.8 ± 14.4	83.1 ± 17.2	0.106
MVR (%)	48.6 ± 0.4	48.5 ± 0.5	48.5 ± 0.4	48.7 ± 0.4**	48.7 ± 0.3	48.7 ± 0.4**	0.001
Height (cm)	142.8 ± 16.1	138.3 ± 17.3	140.4 ± 16.5	145.4 ± 15.6**	145.7 ± 13.7	148.7 ± 13.4*^,^**	<0.001
Body weight (kg)	39.0 ± 14.5	28.4 ± 9.1	33.3 ± 10.5	42.4 ± 12.6*^,^**	47.1 ± 11.5*^,^**	59.0 ± 16.7*^,^**^,^***^,^****	<0.001
Rohrer index (kg/m^3^)	129.5 ± 22.7	105.2 ± 9.6	117.5 ± 10.0*	134.3 ± 11.1*^,^**	150.4 ± 11.0*^,^**^,^***	176.3 ± 22.5*^,^**^,^***^,^****	<0.001
POW (%)	2.9 ± 18.2	−16.5 ± 5.6	−7.2 ± 6.6*	7.0 ± 8.1*^,^**	20.6 ± 7.2*^,^**^,^***	41.6 ± 17.6*^,^**^,^***^,^****	<0.001
BMI (kg/m^2^)	18.5 ± 3.8	14.4 ± 1.2	16.4 ± 1.7*	19.5 ± 2.3*^,^**	21.8 ± 1.6*^,^**^,^***	26.2 ± 3.7*^,^**^,^***^,^****	<0.001
Body fat mass (kg)	8.8 ± 7.3	2.2 ± 1.4	5.2 ± 2.8*	10.0 ± 4.3*^,^**	14.0 ± 4.1*^,^**^,^***	23.6 ± 9.5*^,^**^,^***^,^****	<0.001
Lean body mass (kg)	30.3 ± 9.0	26.2 ± 8.2	28.2 ± 8.4	32.4 ± 9.1*^,^**	33.1 ± 8.0*^,^**	35.3 ± 8.9*^,^**	<0.001

CAVI, cardio-ankle vascular index; SBP, systolic blood pressure; DBP, diastolic blood pressure; MBP, mean blood pressure; PP, pulse pressure; HR, heart rate; MVR, muscular vessel ratio; POW, percentage of overweight; BMI, body mass index.

* *p* < 0.05 vs. underweight.

** *p* < 0.05 vs. −normal.

*** *p* < 0.05 vs. +normal.

**** *p* < 0.05 vs. overweight.

**Table 3:** Cardio-ankle vascular index of participants by percentage of overweight classification (*n* = 590).

POW categories	Total	Underweight	Normal	Mild obesity	Moderate obesity	Severe obesity	*p* value
POW (%)		POW ≤ −20	−20 < POW < + 20	+20 ≤ POW < +30	+30 ≤ POW < +50	+50 ≤ POW	
POW (%)	2.6 ± 17.9	−22.4 ± 2.8	−2.1 ± 9.7	23.6 ± 2.6	37.8 ± 6.0	62.2 ± 13.2	<0.001
*N*	590	20	491	29	33	17	-
CAVI	4.7 ± 0.6	5.1 ± 0.7	4.7 ± 0.6*	4.5 ± 0.5*	4.4 ± 0.5*	4.1 ± 0.9*^,^**	<0.001
Age, *n* (%)	10.5 ± 2.6	11.7 ± 2.5	10.4 ± 2.7	11.1 ± 2.3	10.6 ± 2.0	11.8 ± 2.6	0.027
Female, *n* (%)	301 (51)	11 (55.0)	253 (51.5)	18 (62.1)	13 (39.4)	6 (35.3)	0.282
SBP (mmHg)	116.9 ± 10.5	115.1 ± 10.0	116.1 ± 10.1	121.1 ± 8.4	120.1 ± 10.5	128.9 ± 13.4*^,^**^,^****	<0.001
DBP (mmHg)	68.7 ± 6.7	65.4 ± 5.7	68.6 ± 6.6	70.5 ± 6.4	69.4 ± 7.1	71.2 ± 8.5	0.045
MBP (mmHg)	84.8 ± 7.1	82.0 ± 6.5	84.4 ± 7.0	87.4 ± 6.2	86.3 ± 7.2	90.4 ± 9.4*^,^**	<0.001
PP (mmHg)	48.2 ± 8.3	50.0 ± 7.7	47.4 ± 8.0	50.6 ± 7.3	50.7 ± 9.1	57.7 ± 9.3*^,^**^,^***^,^****	<0.001
HR (bpm)	78.9 ± 14.6	72.6 ± 12.3	78.5 ± 14.4	83.1 ± 20.6	81.1 ± 12.6	84.8 ± 12.6	0.044
MVR (%)	48.6 ± 0.4	48.7 ± 0.3	48.6 ± 0.4	48.6 ± 0.3	48.7 ± 0.4	48.7 ± 0.4	0.148
Height (cm)	142.5 ± 16.1	151.3 ± 13.2	141.6 ± 16.4	144.8 ± 14.1	144.8 ± 12.1	151.9 ± 15.5	0.005
Body weight (kg)	38.7 ± 14.4	34.1 ± 8.1	36.2 ± 12.3	48.1 ± 13.3*^,^**	53.3 ± 12.7*^,^**	71.4 ± 19.0*^,^**^,^***^,^****	<0.001
Rohrer index (kg/m^3^)	129.1 ± 22.3	96.9 ± 6.9	123.5 ± 12.7*	154.8 ± 5.9*^,^**	172.7 ± 9.4*^,^**^,^***	200.3 ± 19.6*^,^**^,^***^,^****	<0.001
BMI (kg/m^2^)	18.4 ± 3.8	14.6 ± 1.3	17.4 ± 2.5*	22.4 ± 2.0*^,^**	25.0 ± 2.0*^,^**^,^***	30.3 ± 3.2*^,^**^,^***^,^****	<0.001
BIA *n* = 561	561	19	463	29	33	17	-
Body fat percentage (%)	20.0 ± 9.6	11.3 ± 5.9	17.6 ± 6.9*	30.3 ± 4.7*^,^**	36.8 ± 4.3*^,^**^,^***	45.0 ± 6.8*^,^**^,^***^,^****	<0.001
Body fat mass (kg)	8.8 ± 7.3	3.9 ± 2.2	6.9 ± 4.5	14.9 ± 5.7*^,^**	19.8 ± 5.9*^,^**^,^***	32.4 ± 11.0*^,^**^,^***^,^****	<0.001
Lean body mass (kg)	30.3 ± 9.0	29.6 ± 7.0	29.6 ± 8.9	33.2 ± 8.3	33.5 ± 7.6	39.0 ± 10.4*^,^**	<0.001

POW, percentage of overweight; CAVI, cardio-ankle vascular index; SBP, systolic blood pressure; DBP, diastolic blood pressure; MBP, mean blood pressure; PP, pulse pressure; HR, heart rate; MVR, muscular vessel ratio; BMI, body mass index; BIA, bioelectrical impedance analysis.

* *p* < 0.05 vs. underweight.

** *p* < 0.05 vs. normal.

*** *p* < 0.05 vs. mild obesity.

**** *p* < 0.05 vs. moderate obesity.

**Table 4:** Cardio-ankle vascular index of participants by body mass index classification (*n* = 590).

Body mass index categories	Total	Underweight	Normal weight	Overweight to obesity	*p* value
Body mass index (kg/m^2^)		Body mass index < 18.5	18.5 ≤ Body mass index < 25	25 ≤ Body mass index	
Body mass index (kg/m^2^)	18.4 ± 3.8	16.0 ± 1.4	21.0 ± 1.7	28.3 ± 3.1	<0.001
*N*	590	360	194	36	-
CAVI	4.7 ± 0.6	4.8 ± 0.6	4.7 ± 0.6	4.3 ± 0.7*^,^**	<0.001
Age, *n* (%)	10.5 ± 2.6	9.5 ± 2.5	11.9 ± 2.1*	12.4 ± 1.9*	<0.001
Female, *n* (%)	301 (51.0)	177 (49.2)	108 (55.7)	16 (44.4)	0.248
SBP (mmHg)	116.9 ± 10.5	114.0 ± 9.6	120.2 ± 9.6*	127.5 ± 11.5*^,^**	<0.001
DBP (mmHg)	68.7 ± 6.7	68.2 ± 6.7	69.4 ± 6.1	70.7 ± 8.4	0.029
MBP (mmHg)	84.8 ± 7.1	83.5 ± 7.0	86.3 ± 6.4*	89.6 ± 8.7*^,^**	<0.001
PP (mmHg)	48.2 ± 8.3	46.0 ± 7.4	50.8 ± 8.0*	56.9 ± 8.3*^,^**	<0.001
HR (bpm)	78.9 ± 14.6	80.1 ± 13.8	76.0 ± 14.5*	82.1 ± 20.0	0.003
MVR (%)	48.6 ± 0.4	48.5 ± 0.4	48.8 ± 0.3*	48.9 ± 0.3*	<0.001
Height (cm)	142.5 ± 16.1	136.5 ± 15.5	151.4 ± 11.9*	155.3 ± 11.0*	<0.001
Body weight (kg)	38.7 ± 14.4	30.5 ± 8.7	48.3 ± 8.5*	68.7 ± 13.6*^,^**	<0.001
Rohrer index (kg/m^3^)	129.1 ± 22.3	118.2 ± 12.4	139.4 ± 16.6*	182.9 ± 22.5*^,^**	<0.001
POW (%)	2.6 ± 17.9	−6.8 ± 8.4	11.6 ± 12.8*	47.6 ± 17.5*^,^**	<0.001
BIA *n* = 561	561	337	188	36	
Body fat percentage (%)	20.0 ± 9.6	14.3 ± 4.9	26.2 ± 6.3*	41.1 ± 6.9*^,^**	<0.001
Body fat mass (kg)	8.8 ± 7.3	4.5 ± 2.3	12.6 ± 3.7*	28.4 ± 8.7*^,^**	<0.001
Lean body mass (kg)	30.2 ± 9.0	26.2 ± 7.5	35.7 ± 7.2*	40.2 ± 8.0*^,^**	<0.001

CAVI, cardio-ankle vascular index; SBP, systolic blood pressure; DBP, diastolic blood pressure; MBP, mean blood pressure; PP, pulse pressure; HR, heart rate; MVR, muscular vessel ratio; POW, percentage of overweight; BIA, bioelectrical impedance analysis.

* *p* < 0.05 vs. underweight.

** *p* < 0.05 vs. normal weight.

In addition, as shown in Table 5, CAVI was positively correlated with age, MVR, height, and lean body mass (*R* = 0.18, *R* = 0.15, *R* = 0.16, and *R* = 0.10, respectively; *p* < 0.05 for all), and was negatively correlated with HR, Rohrer index, POW, BMI, body fat percentage, and body fat mass (*R* = −0.14, *R* = −0.33, *R* = −0.30, *R* = −0.19, *R* = −0.23 and *R* = −0.14, respectively; *p* < 0.01, for all). In contrast, there were no significant correlations between CAVI and SBP or body weight.

**Table 5:** Simple correlation analyses.

Parameters	CAVI	Age	SBP	MBP	PP	HR	MVR	Height	Body weight	Rohrer index	POW	BMI	BFP	BFM	LBM
CAVI	-	0.18**	−0.06	−0.05	−0.05	−0.14**	0.15**	0.16**	−0.01	−0.33**	−0.30**	−0.19**	−0.23**	−0.14**	0.10*
Age	0.18**	-	0.48**	0.35**	0.45**	−0.29**	0.71**	0.90**	0.76**	−0.04	0.06	0.46**	0.30**	0.46**	0.85**
SBP	−0.06	0.48**	-	0.87**	0.77**	0.01	0.43**	0.53**	0.56**	0.16**	0.25**	0.44**	0.32**	0.43**	0.56**
MBP	−0.05	0.35**	0.87**	-	0.36**	0.12**	0.31**	0.38**	0.40**	0.12**	0.18**	0.32**	0.26**	0.33**	0.40**
PP	−0.05	0.45**	0.77**	0.36**	-	−0.14**	0.40**	0.50**	0.54**	0.15**	0.24**	0.41**	0.27**	0.40**	0.55**
HR	−0.14**	−0.29**	0.01	0.12**	−0.14**	-	−0.24**	−0.27**	−0.18**	0.11**	0.09*	−0.05	0.07	−0.01	−0.27**
MVR	0.15**	0.71**	0.43**	0.31**	0.40**	−0.24**	-	0.80**	0.68**	−0.05	0.06	0.40**	0.27**	0.41**	0.76**
Height	0.16**	0.90**	0.53**	0.38**	0.50**	−0.27**	0.80**	-	0.85**	−0.05	0.09*	0.50**	0.32**	0.51**	0.95**
Body weight	−0.01	0.76**	0.56**	0.40**	0.54**	−0.18**	0.68**	0.85**	-	0.45**	0.57**	0.87**	0.69**	0.86**	0.91**
Rohrer index	−0.33**	−0.04	0.16**	0.12**	0.15**	0.11**	−0.05	−0.05	0.45**	-	0.98**	0.83**	0.83**	0.75**	0.12**
POW	−0.30**	0.06	0.25**	0.18**	0.24**	0.09*	0.06	0.09*	0.57**	0.98**	-	0.89**	0.85**	0.81**	0.26**
BMI	−0.19**	0.46**	0.44**	0.32**	0.41**	−0.05	0.40**	0.50**	0.87**	0.83**	0.89**	-	0.90**	0.95**	0.62**
BFP	−0.23**	0.30**	0.32**	0.26**	0.27**	0.07	0.27**	0.32**	0.69**	0.83**	0.85**	0.90**	-	0.91**	0.37**
BFM	−0.14**	0.46**	0.43**	0.33**	0.40**	−0.01	0.41**	0.51**	0.86**	0.75**	0.81**	0.95**	0.91**	-	0.58**
LBM	0.10*	0.85**	0.56**	0.40**	0.55**	−0.27**	0.76**	0.95**	0.91**	0.12**	0.26**	0.62**	0.37**	0.58**	-

CAVI, cardio-ankle vascular index; SBP, systolic blood pressure; MBP, mean blood pressure; PP, pulse pressure; HR, heart rate; MVR, muscular vessel ratio; POW, percentage of overweight; BMI, body mass index, BFP, body fat percentage, BFM, body fat mass, LBM, lean body mass.

* *p* < 0.05.

** *p* < 0.01.

Furthermore, multiple linear regression analyses adjusted for age, sex, blood pressure, HR and MVR showed that Rohrer index, POW, BMI, body fat percentage, body fat mass and lean body mass were independent predictors of CAVI (Table 6).

**Table 6:** Linear regression analyses of cardio-ankle vascular index.

Factor	Simple regression analysis		Model 1		Model 2		Model 3	
	*β*	*p* value	*β*	*p* value	*β*	*p* value	*β*	*p* value
Age	0.18	<0.01	-	-	-	-	-	-
Female	−0.10	0.02	-	-	-	-	-	-
Systolic blood pressure	−0.06	0.13	−0.21	<0.01	-	-	-	-
Mean blood pressure	−0.05	0.20	−0.14	<0.01	-	-	-	-
Pulse pressure	−0.05	0.22	−0.19	<0.01	-	-	-	-
Heart rate	−0.14	<0.01	−0.09	0.04	-	-	-	-
Muscular vessel ratio	0.15	<0.01	0.03	0.56	0.06	0.29	-	-
Rohrer index	−0.32	<0.01	−0.32	<0.01	−0.29	<0.01	−0.29	<0.01
Percentage of overweight	−0.30	<0.01	−0.32	<0.01	−0.29	<0.01	−0.29	<0.01
Body mass index	−0.19	<0.01	−0.35	<0.01	−0.31	<0.01	−0.31	<0.01
Body fat percentage	−0.23	<0.01	−0.30	<0.01	−0.27	<0.01	−0.28	<0.01
Body fat mass	−0.14	<0.01	−0.28	<0.01	−0.24	<0.01	−0.25	<0.01
Lean body mass	0.10	0.02	−0.31	<0.01	−0.23	0.01	−0.30	0.01

Model 1: adjusted for age and sex. Model 2: adjusted for age, sex, systolic blood pressure and heart rate. Model 3: adjusted for age, sex, systolic blood pressure, heart rate and muscular vessel ratio.

## Discussion

To the best of our knowledge, the present study is the first to investigate the associations between multiple obesity parameters (Rohrer index, body fat percentage, body fat mass, POW, BMI) and CAVI in healthy children and adolescents. The main findings of this study were that: (A) manually measured and CAVI-based vessel lengths showed excellent concordance, suggesting the accuracy of CAVI; (B) CAVI was not only negatively correlated with obesity parameters, and but also positively correlated with age, height, and MVR, suggesting that CAVI reflects muscular vessel components; and (C) even after adjusting for possible obesity-related factors including age, sex, blood pressure and MVR, all obesity parameters itself remained independent predictors of CAVI in healthy children and adolescents.

CAVI measurement is a simple, non-invasive procedure that can be completed in a short time. Accurate measurements are possible if the child remains still and quiet to avoid interference with pulse waves and heart sound recordings. In children, it is important to select the appropriate cuff size, considering the individual variations in arm circumference. In addition, the length of the blood vessel is an important factor in determining the pulse wave velocity in CAVI measurement, and the current formula can be employed to calculate the length of the blood vessel using the height of the child. The present study demonstrated successful CAVI application in children and adolescents in terms of smooth measurements and accurate vessel length estimation.

Our study in children revealed a significant negative correlation between CAVI and multiple obesity parameters, including Rohrer index, body fat indices measured by BIA, POW, and BMI. Similar to our findings, CAVI has been reported to be negatively correlated with BMI in adults (6, 7, 29). Additionally, in children, both CAVI and pulse wave velocity have been shown to have a negative correlation with BMI (30–32). Although CAVI is an indicator that reflects arterial stiffness, most studies have shown a negative correlation between CAVI and BMI, indicating that lower CAVI is associated with obesity. Unlike previous CAVI studies which focused on BMI only (7, 30), our study also investigated the other obesity parameters (i.e., Rohrer index, POW, body fat percentage and body fat mass determined by BIA) in children. We firstly used the Rohrer index and POW in the present study, which is known to be appropriate for assessing childhood obesity (16, 17). We further measured body fat percentage base on the accurate BIA method, which is superior to BMI or Rohrer index to evaluate objective obesity (18, 19). As a result, not only BMI but also other obesity parameters showed a negative correlation with CAVI, and these results lead to establish a clear relationship between obesity and reduced CAVI.

We could not fully explain the reason for the “obesity paradox”; however, possible explanations are as follows: first, it has been reported that obesity induces adaptive physiological changes (e.g., vasodilation, increased blood volume and cardiac output, and changes in metabolic and hemodynamic demands) (30–32). Second, CAVI measures arterial properties across different vessel types, including the aorta, an elastic artery, and the femoral and tibial arteries, which are muscular vessels (20). Mileva et al. reported that the vasoreactivity for antihypertensive drugs was different in ascending aorta and descending aorta using computed tomography (33). These results suggest that there might be differences in response for antihypertensive drugs between the elastic and muscular arteries. We have firstly examined MVR, which is the ratio of elastic and muscular blood vessels, and found that CAVI especially reflects muscular vessel components.

Czippelova et al. reported that the diameter of the smaller peripheral resistance arteries is under the control of the sympathetic nervous system and sympatho-vagal balance. They also reported that obesity is associated with decreased sympathetic nervous activity and lower systemic vascular resistance, leading to low CAVI (32). CAVI was negatively correlated with heart rate, and might be affected by the impaired sympathetic nervous system in obese people. However, not only impaired sympathetic nerve activity (32, 34, 35), but also sympathetic nerve activity (36, 37) and shift in sympatho-vagal balance have previously been reported in obese people. In addition, it has been reported that arterial stiffness of muscular vessel (i.e., iliofemoral artery Beta) (38, 39) is lower in the Watanabe heritable hyperlipidemic rabbits than in normal rabbits (39, 40). The authors consider that arterial stiffness of muscular vessels might decrease at first stage where infiltration of lipids might soften the arterial wall. Third, perivascular fat in obese individuals may act as buffer, reducing pulse wave conduction and potentially lowering CAVI. In addition, in children during the growth period, fat content increases along with body development. Fourth, fat distribution and metabolic state may impact CAVI. Christakoudi et al. reported that obese individuals without abdominal fat can maintain a healthy metabolic state (41). These results support the hypothesis that the systemic accumulation of adipose tissue *per se* leads to decreased arterial stiffness in obese individuals without metabolic disorders; thus, adipose tissue in healthy children and adolescents might be associated with a healthy metabolic state.

### Clinical implications

CAVI can be measured in children in the same way it is measured in adults. However, in cases of childhood obesity, caution is needed because arterial stiffness can be underestimated.

### Study limitations

There are several limitations to our study. First, the present study was cross-sectional of healthy children, did not include participant with severe obesity, and did not follow-up. Thus, the relationship between childhood obesity and future cardiovascular and cerebrovascular disease is unknown. Second, we did not measure abdominal circumference, body shape index (42), laboratory data (such as blood glucose and lipid profile, which are components of metabolic syndrome), or other CAVI parameters (e.g., CAVI_0_) (43). Third, we manually evaluated vessel length using a tape measure, rather than using more precise methods like magnetic resonance imaging or computed tomography and did not measure vessel length and diameter. Fourth, we assessed only total body fat mass and body fat percentage, without considering the distribution of adipose tissue. Additionally, we did not measure visceral fat, epicardial fat, or subcutaneous fat. Fifth, our study was conducted with children and adolescents in the pubertal period. Given that development during this period significantly influences various physiological characteristics, including body composition and autonomic nervous system activity, another limitation is the lack of information on the stage of pubertal development (e.g., Tanner score) (32).

## Conclusion

Even after consideration of possible obesity related factors (age, sex, blood pressure, and heart rate, MVR, and etc.), all obesity parameters (POW, Rohrer index, BMI, body fat percentage, and body fat mass) are negatively and independently associated with CAVI in healthy children. In cases of childhood obesity, caution is needed because arterial stiffness can be underestimated.

## Data Availability

The raw data supporting the conclusions of this article will be made available by the authors, without undue reservation.

## References

[1] Tavolinejad H Erten O Maynard H Chirinos JA. Prognostic value of cardio-ankle vascular index for cardiovascular and kidney outcomes: systematic review and meta-analysis. JACC Adv. (2024) 3(7):101019. 10.1016/j.jacadv.2024.101019 39130005 PMC11312768

[2] Sato Y Yoshihisa A Ichijo Y Watanabe K Hotsuki Y Kimishima Y Cardio-ankle vascular index predicts post-discharge stroke in patients with heart failure. J Atheroscler Thromb. (2021) 28(7):766–75. 10.5551/jat.58727 32981919 PMC8265923

[3] Watanabe K Yoshihisa A Sato Y Hotsuki Y Anzai F Ichijo Y Cardio-ankle vascular index reflects impaired exercise capacity and predicts adverse prognosis in patients with heart failure. Front Cardiovasc Med. (2021) 8:631807. 10.3389/fcvm.2021.631807 33869301 PMC8044779

[4] Shimizu T Sakuma Y Muto Y Anzai F Kimishima Y Sato Y Impact of cardio-ankle vascular index on future cancer in patients with coronary artery disease. Circ Rep. (2024) 6(9):372–80. 10.1253/circrep.CR-24-0070 39262639 PMC11383543

[5] Okamoto Y Miyoshi T Ichikawa K Takaya Y Nakamura K Ito H. Cardio-ankle vascular index as an arterial stiffness marker improves the prediction of cardiovascular events in patients without cardiovascular diseases. J Cardiovasc Dev Dis. (2022) 9(11):368. 10.3390/jcdd9110368 36354767 PMC9698795

[6] Sugiura T Dohi Y Takagi Y Yoshikane N Ito M Suzuki K Relationships of obesity-related indices and metabolic syndrome with subclinical atherosclerosis in middle-aged untreated Japanese workers. J Atheroscler Thromb. (2020) 27(4):342–52. 10.5551/jat.50633 31462618 PMC7192820

[7] Nagayama D Imamura H Sato Y Yamaguchi T Ban N Kawana H Inverse relationship of cardioankle vascular index with BMI in healthy Japanese subjects: a cross-sectional study. Vasc Health Risk Manag. (2017) 13:1–9. 10.2147/VHRM.S119646 28053538 PMC5189698

[8] Cote AT Harris KC Panagiotopoulos C Sandor GG Devlin AM. Childhood obesity and cardiovascular dysfunction. J Am Coll Cardiol. (2013) 62(15):1309–19. 10.1016/j.jacc.2013.07.042 23954339

[9] Maeda M Maeda T Ihara K. Secular trends in obesity and serum lipid values among children in Oita city, Japan, during a 27-year period. J Atheroscler Thromb. (2022) 29(12):1709–26. 10.5551/jat.63056 35095055 PMC9879566

[10] Nakashima Y Chen YX Kinukawa N Sueishi K. Distributions of diffuse intimal thickening in human arteries: preferential expression in atherosclerosis-prone arteries from an early age. Virchows Arch. (2002) 441(3):279–88. 10.1007/s00428-002-0605-1 12242525

[11] Brown CL Halvorson EE Cohen GM Lazorick S Skelton JA. Addressing childhood obesity: opportunities for prevention. Pediatr Clin North Am. (2015) 62(5):1241–61. 10.1016/j.pcl.2015.05.013 26318950 PMC4555982

[12] Morita N Kambayashi I Okuda T Oda S Takada S Nakajima T Inverse relationship between sleep duration and cardio-ankle vascular index in children. J Atheroscler Thromb. (2017) 24(8):819–26. 10.5551/jat.36517 27904026 PMC5556189

[13] Nakagawa R Kuwata S Kurishima C Saiki H Iwamoto Y Sugimoto M Arterial stiffness in patients after Kawasaki disease without coronary artery involvement: assessment by performing brachial ankle pulse wave velocity and cardio-ankle vascular index. J Cardiol. (2015) 66(2):130–4. 10.1016/j.jjcc.2014.10.003 25458191

[14] Sugiura R Murata M. Problems with body mass index as an index to evaluate physical status of children in puberty. Pediatr Int. (2011) 53(5):634–42. 10.1111/j.1442-200X.2010.03312.x 21159030

[15] Dobashi K. Evaluation of obesity in school-age children. J Atheroscler Thromb. (2016) 23(1):32–8. 10.5551/jat.29397 26510873

[16] Kataoka K. Indices of obesity derived from body weight and height. Nihon Rinsho Jpn J Clin Med. (1995) 53(Suppl):147–53. 7563679

[17] Nishida M Funahashi T. Validity of indices (BMI, Rohrer index, Broca method) for assessment of obesity. Nihon Rinsho. (2009) 67(2):301–6. 19202903

[18] Messner A Nairz J Kiechl S Winder B Pechlaner R Geiger R Comparison of body mass index and fat mass index to classify body composition in adolescents-the EVA4YOU study. Eur J Pediatr. (2024) 183(5):2203–14. 10.1007/s00431-024-05474-x 38386029 PMC11035421

[19] Guida B Pietrobelli A Trio R Laccetti R Falconi C Perrino NR Body mass index and bioelectrical vector distribution in 8-year-old children. Nutr Metab Cardiovasc Dis. (2008) 18(2):133–41. 10.1016/j.numecd.2006.08.008 17307345

[20] Shirai K Utino J Otsuka K Takata M. A novel blood pressure-independent arterial wall stiffness parameter; cardio-ankle vascular index (CAVI). J Atheroscler Thromb. (2006) 13(2):101–7. 10.5551/jat.13.101 16733298

[21] Horikoshi Y Katsuda SI Fujikura Y Hazama A Shimura H Shimizu T Opposing responses of the calcium channel blocker nicardipine to vascular stiffness in the elastic and muscular arteries in rabbits. J Atheroscler Thromb. (2021) 28(12):1340–8. 10.5551/jat.60848 33746145 PMC8629710

[22] Yamamoto T Shimizu K Takahashi M Tatsuno I Shirai K. The effect of nitroglycerin on arterial stiffness of the aorta and the femoral-tibial arteries. J Atheroscler Thromb. (2017) 24(10):1048–57. 10.5551/jat.38646 28331159 PMC5656767

[23] Yamada Y Nishizawa M Uchiyama T Kasahara Y Shindo M Miyachi M Developing and validating an age-independent equation using multi-frequency bioelectrical impedance analysis for estimation of appendicular skeletal muscle mass and establishing a cutoff for sarcopenia. Int J Environ Res Public Health. (2017) 14(7):809. 10.3390/ijerph14070809 28753945 PMC5551247

[24] Dobashi K Takahashi K Nagahara K Tanaka D Itabashi K. Evaluation of hip/height(P) ratio as an index for adiposity and metabolic complications in obese children: comparison with waist-related indices. J Atheroscler Thromb. (2017) 24(1):47–54. 10.5551/jat.35311 27298049 PMC5225132

[25] Tominaga K Kurata JH Chen YK Fujimoto E Miyagawa S Abe I Prevalence of fatty liver in Japanese children and relationship to obesity. An epidemiological ultrasonographic survey. Dig Dis Sci. (1995) 40(9):2002–9. 10.1007/BF02208670 7555456

[26] WHO Expert Consultation. Appropriate body-mass index for Asian populations and its implications for policy and intervention strategies. Lancet. (2004) 363(9403):157–63. 10.1016/S0140-6736(03)15268-3 14726171

[27] Examination Committee of Criteria for ‘Obesity Disease’ in Japan, Japan Society for the Study of Obesity. New criteria for ‘obesity disease’ in Japan. Circ J. (2002) 66(11):987–92. 10.1253/circj.66.987 12419927

[28] Cole TJ Lobstein T. Extended international (IOTF) body mass index cut-offs for thinness, overweight and obesity. Pediatr Obes. (2012) 7(4):284–94. 10.1111/j.2047-6310.2012.00064.x 22715120

[29] Park HE Choi SY Kim HS Kim MK Cho SH Oh BH. Epicardial fat reflects arterial stiffness: assessment using 256-slice multidetector coronary computed tomography and cardio-ankle vascular index. J Atheroscler Thromb. (2012) 19(6):570–6. 10.5551/jat.12484 22472214

[30] Philip R Alpert BS Schwingshackl A Huang X Blakely D Rovnaghi CR Inverse relationship between cardio-ankle vascular index and body mass index in healthy children. J Pediatr. (2015) 167(2):361–5.e1. 10.1016/j.jpeds.2015.04.042 26003881

[31] Dangardt F Osika W Volkmann R Gan LM Friberg P. Obese children show increased intimal wall thickness and decreased pulse wave velocity. Clin Physiol Funct Imaging. (2008) 28(5):287–93. 10.1111/j.1475-097X.2008.00806.x 18476996

[32] Czippelova B Turianikova Z Krohova J Wiszt R Lazarova Z Pozorciakova K Arterial stiffness and endothelial function in young obese patients—vascular resistance matters. J Atheroscler Thromb. (2019) 26(11):1015–25. 10.5551/jat.47530 30930343 PMC6845697

[33] Mileva N Panayotov P Hristova I Koleva G Georgieva D Ivanova R Impact of renin-angiotensin system targeted therapy on aortic elastic properties assessed by computed tomography. Int J Cardiol Heart Vasc. (2024) 55:101562. 10.1016/j.ijcha.2024.101562 39649025 PMC11625146

[34] Peterson HR Rothschild M Weinberg CR Fell RD McLeish KR Pfeifer MA. Body fat and the activity of the autonomic nervous system. N Engl J Med. (1988) 318(17):1077–83. 10.1056/NEJM198804283181701 3352710

[35] Nagai N Matsumoto T Kita H Moritani T. Autonomic nervous system activity and the state and development of obesity in Japanese school children. Obes Res. (2003) 11(1):25–32. 10.1038/oby.2003.6 12529482

[36] Cvijetic S Macan J Boschiero D Ilich JZ. Body fat and muscle in relation to heart rate variability in young-to-middle age men: a cross sectional study. Ann Hum Biol. (2023) 50(1):108–16. 10.1080/03014460.2023.2180089 36786451

[37] Plaza-Florido A Migueles JH Mora-Gonzalez J Molina-Garcia P Rodriguez-Ayllon M Cadenas-Sanchez C The role of heart rate on the associations between body composition and heart rate variability in children with overweight/obesity: the activebrains project. Front Physiol. (2019) 10:895. 10.3389/fphys.2019.00895 31379602 PMC6646801

[38] Takahashi K Yamamoto T Tsuda S Okabe F Shimose T Tsuji Y Coefficients in the CAVI equation and the comparison between CAVI with and without the coefficients using clinical data. J Atheroscler Thromb. (2019) 26(5):465–75. 10.5551/jat.44834 30518727 PMC6514175

[39] Katsuda SI Horikoshi Y Shiomi M Kitajima S Ito T Hazama A Arterial stiffness of the aorta and iliofemoral artery and their responses to nitroglycerin administration in myocardial infarction-prone Watanabe heritable hyperlipidemic rabbits. J Hypertens. (2024) 42(3):441–9. 10.1097/HJH.0000000000003608 37937516 PMC10842652

[40] Miyata M. Basic research sheds light on the aspect of cardio-ankle vascular index (CAVI) including elastic and muscular arteries. J Atheroscler Thromb. (2021) 28(6):588–9. 10.5551/jat.ED147 33041314 PMC8219537

[41] Christakoudi S Tsilidis KK Muller DC Freisling H Weiderpass E Overvad K A body shape index (ABSI) achieves better mortality risk stratification than alternative indices of abdominal obesity: results from a large European cohort. Sci Rep. (2020) 10(1):14541. 10.1038/s41598-020-71302-5 32883969 PMC7471961

[42] Nagayama D Watanabe Y Yamaguchi T Maruyama M Saiki A Shirai K New index of abdominal obesity, a body shape index, is BMI-independently associated with systemic arterial stiffness in real-world Japanese population. Int J Clin Pharmacol Ther. (2020) 58(12):709–17. 10.5414/CP203778 32831165

[43] Takahashi K Yamamoto T Tsuda S Maruyama M Shirai K. The background of calculating CAVI: lesson from the discrepancy between CAVI and CAVI(0). Vasc Health Risk Manag. (2020) 16:193–201. 10.2147/VHRM.S223330 32547046 PMC7251085

