# Supplementary material for: Associations between obesity and arterial stiffness assessed by cardio-ankle vascular index in healthy children and adolescents
Source: Front Cardiovasc Med. 2025 Sep 9;12:1633849. doi: 10.3389/fcvm.2025.1633849 (PMC12454398; doi:10.3389/fcvm.2025.1633849)
Supplement: Supplementary file 1 [file Datasheet1.docx]

**Supplementary Table 1a. Cardio-ankle vascular index of participants by Rohrer index classification (Male, *n* = 289)**

| **Rohrer index categories** | **Total** | **Lean** | **Moderately lean** | **Normal** | **Moderately obese** | **Obese** | ***p* value** |
| --- | --- | --- | --- | --- | --- | --- | --- |
| **Rohrer index (kg/m^3^)** |  | Rohrer index  ˂ 100 | 100 ≤ Rohrer index ˂ 115 | 115 ≤ Rohrer index ˂ 145 | 145 ≤ Rohrer index ˂ 160 | 160 ≤ Rohrer index |  |
| **Rohrer index (kg/m^3^)** | 129.5 ± 23.5 | 103.1 ± 15.8 | 107.8 ± 4.4 | 128.0 ± 8.2 | 148.8 ± 6.9 | 179.3 ± 20.0 | <0.001 |
| ***n*** | 289 | 17 | 63 | 153 | 24 | 32 |  |
| **CAVI** | 4.8 ± 0.7 | 5.1 ± 0.7 | 5.1 ± 0.6 | 4.7 ± 0.6 ^**^ | 4.5 ± 0.5 ^*, **^ | 4.4 ± 0.7 ^*, **^ | <0.001 |
| **Age, *n* (%)** | 10.4 ± 2.7 | 12.1 ± 2.2 | 11.4 ± 2.4 | 9.8 ± 2.7 ^*, **^ | 9.8 ± 2.3 ^*^ | 11.0 ± 2.4 | <0.001 |
| **SBP (mmHg)** | 117.6 ± 11.0 | 122.7 ± 8.4 | 115.9 ± 11.5 | 116.4 ± 10.5 | 117.9 ± 8.7 | 123.5 ± 12.9 ^**, †^ | 0.002 |
| **DBP (mmHg)** | 68.4 ± 6.7 | 69.3 ± 6.9 | 68.4 ± 6.8 | 68.1 ± 6.5 | 67.7 ± 6.5 | 69.3 ± 7.9 | 0.850 |
| **MBP (mmHg)** | 84.8 ± 7.3 | 87.1 ± 7.0 | 84.2 ± 7.6 | 84.2 ± 6.9 | 84.4 ± 6.5 | 87.4 ± 8.5 | 0.129 |
| **PP (mmHg)** | 49.2 ± 8.9 | 53.4 ± 5.3 | 47.4 ± 8.7 | 48.3 ± 8.8 | 50.2 ± 7.0 | 54.2 ± 10.5 ^**, †^ | <0.001 |
| **HR (bpm)** | 77.8 ± 15.3 | 70.1 ± 9.0 | 72.4 ± 12.8 | 78.7 ± 15.6 | 84.1 ± 21.2 ^*, **^ | 83.0 ± 11.3 ^**^ | <0.001 |
| **MVR (%)** | 48.6 ± 0.4 | 49.0 ± 0.3 | 48.7 ± 0.4 | 48.5 ± 0.5 ^*, **^ | 48.6 ± 0.3 ^*^ | 48.7 ± 0.4 | <0.001 |
| **Height (cm)** | 142.9 ± 17.7 | 158.1 ± 15.3 | 147.4 ± 15.8 | 138.9 ± 18.3 ^*, **^ | 139.5 ± 14.2 ^*^ | 147.5 ± 14.8 | <0.001 |
| **Body weight (kg)** | 39.2 ± 15.8 | 41.1 ± 10.2 | 35.5 ± 10.7 | 36.0 ± 14.6 | 41.8 ± 13.5 | 59.0 ± 19.1 ^*, **, †, ‡^ | <0.001 |
| **POW (%)** | 3.8 ± 18.9 | -14.3 ± 11.3 | -12.8 ± 3.7 | 1.8 ± 7.6 ^*, **^ | 18.4 ± 7.5 ^*, **, †^ | 44.6 ± 16.3 ^*, **, †, ‡^ | <0.001 |
| **BMI (kg/m^2^)** | 18.5 ± 4.0 | 16.2 ± 2.3 | 15.9 ± 1.5 | 17.8 ± 2.6 ^**^ | 20.8 ± 2.6 ^*, **, †^ | 26.4 ± 3.7 ^*, **, †, ‡^ | <0.001 |
| **BIA *n* = 272** | 272 | 10 | 61 | 147 | 23 | 31 |  |
| **Body fat percentage (%)** | 17.5 ± 10.3 | 7.2 ± 2.3 | 9.1 ± 3.0 | 15.7 ± 5.6 ^*, **^ | 26.5 ± 5.0 ^*, **, †^ | 38.9 ± 6.2 ^*, **, †, ‡^ | <0.001 |
| **Body fat mass (kg)** | 8.0 ± 7.8 | 3.1 ± 1.3 | 3.5 ± 1.9 | 6.3 ± 4.3 ^**^ | 11.7 ± 5.2 ^*, **, †^ | 23.6 ± 10.3 ^*, **, †, ‡^ | <0.001 |
| **Lean body mass (kg)** | 31.6 ± 10.5 | 39.3 ± 8.7 | 32.0 ± 9.2 | 30.1 ± 10.9 | 30.9 ± 8.5 | 36.0 ± 10.5 ^†^ | 0.006 |

CAVI, cardio-ankle vascular index; SBP, systolic blood pressure; DBP, diastolic blood pressure; MBP, mean blood pressure; PP, pulse pressure; HR, heart rate; MVR, muscular vessel ratio.

POW, percentage of overweight; BMI, body mass index; BIA, bioelectrical impedance analysis.

* *p* < 0.05 vs. lean, ** *p* < 0.05 vs. moderately lean, † *p* < 0.05 vs. normal, ‡ *p* < 0.05 vs. moderately obese

**Supplementary Table 1b. Cardio-ankle vascular index of participants by Rohrer index classification (Female, *n* = 289)**

| **Rohrer index categories** | **Total** | **Lean** | **Moderately lean** | **Normal** | **Moderately obese** | **Obese** | ***p* value** |
| --- | --- | --- | --- | --- | --- | --- | --- |
| **Rohrer index (kg/m^3^)** |  | Rohrer index  ˂ 100 | 100 ≤ Rohrer index ˂ 115 | 115 ≤ Rohrer index ˂ 145 | 145 ≤ Rohrer index ˂ 160 | 160 ≤ Rohrer index |  |
| **Rohrer index (kg/m^3^)** | 128.7 ± 21.2 | 97.7 ± 18 | 109.3 ± 4.1 | 127.6 ± 8.6 | 150.4 ± 6.7 | 177.7 ± 22.2 | <0.001 |
| ***n*** | 301 | 7 | 74 | 167 | 28 | 25 | - |
| **CAVI** | 4.6 ± 0.6 | 4.9 ± 0.4 | 4.8 ± 0.6 | 4.7 ± 0.6 | 4.4 ± 0.5 ^**^ | 4.2 ± 0.6 ^**, †^ | <0.001 |
| **Age, *n* (%)** | 10.6 ± 2.6 | 11.3 ± 1.1 | 10.8 ± 2.2 | 10.3 ± 2.8 | 11.4 ± 2.3 | 10.6 ± 2.6 | 0.216 |
| **SBP (mmHg)** | 116.2 ± 9.9 | 113.0 ± 10.7 | 116.2 ± 8.5 | 115.1 ± 10.2 | 120.4 ± 8.9 | 120.0 ± 10.5 | 0.020 |
| **DBP (mmHg)** | 69.1 ± 6.6 | 66.9 ± 6.3 | 69.4 ± 6.3 | 68.5 ± 6.9 | 71.0 ± 6.0 | 70.1 ± 6.0 | 0.294 |
| **MBP (mmHg)** | 84.8 ± 7.0 | 82.2 ± 7.1 | 85.0 ± 6.2 | 84.1 ± 7.4 | 87.4 ± 6.3 | 86.7 ± 6.6 | 0.067 |
| **PP (mmHg)** | 47.2 ± 7.5 | 46.1 ± 7.9 | 46.8 ± 7.4 | 46.6 ± 7.4 | 49.4 ± 6.7 | 49.9 ± 8.6 | 0.127 |
| **HR (bpm)** | 79.9 ± 13.8 | 81.1 ± 11.0 | 81.5 ± 14.9 | 79.4 ± 13.5 | 77.7 ± 13.8 | 80.7 ± 14.0 | 0.738 |
| **MVR (%)** | 48.6 ± 0.4 | 48.7 ± 0.3 | 48.6 ± 0.4 | 48.5 ± 0.4 | 48.7 ± 0.3 | 48.6 ± 0.4 | 0.072 |
| **Height (cm)** | 142.2 ± 14.3 | 149.4 ± 6.9 | 144.3 ± 11.5 | 140.1 ± 15.7 | 147.8 ± 12.7 | 141.9 ± 12.9 | 0.023 |
| **Body weight (kg)** | 38.1 ± 12.9 | 32.7 ± 4.0 | 33.5 ± 7.8 | 36.4 ± 11.8 | 49.6 ± 12.0 ^*, **, †^ | 52.6 ± 17.1 ^*, **, †^ | <0.001 |
| **POW (%)** | 1.4 ± 16.7 | -21.2 ± 2.0 | -13.1 ± 4.1 | -0.007 ± 7.0 ^*, **^ | 19.4 ± 5.8 ^*, **, †^ | 39.9 ± 18.4 ^*, **, †, ‡^ | <0.001 |
| **BMI (kg/m^2^)** | 18.3 ± 3.6 | 14.6 ± 0.5 | 17.9 ± 2.3 | 21.1 ± 5.8 ^*, **^ | 31.5 ± 4.4 ^*, **, †^ | 38.5 ± 7.7 ^*, **, †, ‡^ | <0.001 |
| **BIA *n* = 289** | 289 | 7 | 71 | 160 | 27 | 24 | - |
| **Body fat percentage (%)** | 22.4 ± 8.2 | 14.2 ± 2.0 | 17.1 ± 3.9 | 21.1 ± 5.8 ^*, **^ | 31.5 ± 4.4 ^*, **, †^ | 38.5 ± 7.7 ^*, **, †, ‡^ | <0.001 |
| **Body fat mass (kg)** | 9.5 ± 6.7 | 4.7 ± 1.0 | 6.0 ± 2.5 | 8.3 ± 4.5 ^**^ | 16.3 ± 5.3 ^*, **, †^ | 21.8 ± 10.4 ^*, **, †, ‡^ | <0.001 |
| **Lean body mass (kg)** | 29.0 ± 7.2 | 28.0 ± 3.1 | 27.8 ± 5.3 | 28.2 ± 7.8 | 34.3 ± 6.3 ^**, †^ | 32.1 ± 6.5 | <0.001 |

CAVI, cardio-ankle vascular index; SBP, systolic blood pressure; DBP, diastolic blood pressure; MBP, mean blood pressure; PP, pulse pressure; HR, heart rate; MVR, muscular vessel ratio.

POW, percentage of overweight; BMI, body mass index; BIA, bioelectrical impedance analysis.

* *p* < 0.05 vs. lean, ** *p* < 0.05 vs. moderately lean, † *p* < 0.05 vs. normal, ‡ *p* < 0.05 vs. moderately obese

**Supplementary Table 2a. Cardio-ankle vascular index of participants by body fat percentage classification (Male, *n* = 272)**

| **Body fat percentage** **categories** | **Total** | **Underweight** | **-Normal** | **+Normal** | **Overweight** | **Obese** | ***p* value** |
| --- | --- | --- | --- | --- | --- | --- | --- |
| **Body fat percentage (%)** | 20.0 ± 9.6 | 7.4 ± 3.4 | 14.8 ± 4.6 | 22.9 ± 4.7 | 29.6 ± 3.5 | 39.2 ± 6.2 | < 0.001 |
| ***n*** | 272 | 26 | 127 | 63 | 19 | 37 |  |
| **CAVI** | 4.8 ± 0.7 | 5.1 ± 0.6 | 4.8 ± 0.7 | 4.7 ± 0.5 | 4.7 ± 0.7 | 4.4 ± 0.7 ^*, **^ | 0.001 |
| **Age, *n* (%)** | 10.4 ± 2.7 | 9.6 ± 3.0 | 10.1 ± 2.8 | 10.8 ± 2.6 | 11.4 ± 1.9 | 11.0 ± 2.4 | 0.047 |
| **SBP (mmHg)** | 117.6 ± 11.0 | 111.0 ± 9.4 | 115.5 ± 10.3 | 120.2 ± 10.6 ^*, **^ | 122.1 ± 7.7 ^*^ | 122.6 ± 12.7 ^*, **^ | <0.001 |
| **DBP (mmHg)** | 68.3 ± 6.8 | 64.8 ± 6.2 | 68.3 ± 6.6 | 68.9 ± 7.1 | 69.6 ± 5.0 | 69.5 ± 7.7 | 0.054 |
| **MBP (mmHg)** | 84.7 ± 7.3 | 80.2 ± 6.9 | 84.0 ± 6.9 | 86.0 ± 7.3 ^*^ | 87.1 ± 5.1 ^*^ | 87.2 ± 8.3 ^*^ | <0.001 |
| **PP (mmHg)** | 49.2 ± 8.9 | 46.2 ± 6.1 | 47.2 ± 8.5 | 51.3 ± 9.0 ^**^ | 52.5 ± 6.9 | 53.1 ± 10.2 ^*, **^ | <0.001 |
| **HR (bpm)** | 77.8 ± 15.5 | 75.3 ± 13.3 | 77.6 ± 15.7 | 75.6 ± 13.9 | 74.3 ± 12.7 | 85.5 ± 18.2 ^†^ | 0.016 |
| **MVR (%)** | 48.6 ± 0.4 | 48.5 ± 0.5 | 48.5 ± 0.5 | 48.7 ± 0.4 ^**^ | 48.7 ± 0.3 | 48.7 ± 0.4 | 0.003 |
| **Height (cm)** | 143.1 ± 17.9 | 137.3 ± 18.8 | 139.6 ± 18.6 | 147.2 ± 17.1 | 150.1 ± 13.3 | 148.5 ± 14.6 | 0.002 |
| **Body weight (kg)** | 39.6 ± 16.1 | 27.8 ± 9.8 | 32.9 ± 11.1 | 43.7 ± 13.6 ^*, **^ | 49.4 ± 11.7 ^*, **^ | 58.9 ± 18.3 ^*, **, †^ | <0.001 |
| **Rohrer index (kg/m^3^)** | 129.9 ± 24.0 | 105.0 ± 10.4 | 117.9 ± 10.9 ^*^ | 133.3 ± 9.1 ^*, **^ | 144.1 ± 8.0 ^*, **, †^ | 175.8 ± 20.7 ^*, **, †, ‡^ | <0.001 |
| **POW (%)** | 4.2 ± 19.3 | -16.6 ± 5.3 | -6.2 ± 6.6 ^*^ | 7.6 ± 6.6 ^*, **^ | 16.9 ± 5.2 ^*, **, †^ | 42.0 ± 16.5 ^*, **, †, ‡^ | <0.001 |
| **BMI (kg/m^2^)** | 18.5 ± 4.1 | 14.3 ± 1.2 | 16.3 ± 1.4 ^*^ | 19.5 ± 1.9 ^*, **^ | 21.6 ± 1.4 ^*, **, †^ | 26.0 ± 3.6 ^*, **, †, ‡^ | <0.001 |
| **Body fat mass (kg)** | 8.0 ± 7.8 | 1.6 ± 0.8 | 3.8 ± 1.6 | 8.8 ± 3.2 ^*, **^ | 13.4 ± 3.0 ^*, **, †^ | 22.7 ± 9.8 ^*, **, †, ‡^ | <0.001 |
| **Lean body mass (kg)** | 31.6 ± 10.5 | 26.2 ± 9.1 | 29.1 ± 9.8 | 34.9 ± 10.7 ^*, **^ | 36.0 ± 8.8 ^*^ | 36.1 ± 10.3 ^*, **^ | <0.001 |

CAVI, cardio-ankle vascular index; SBP, systolic blood pressure; DBP, diastolic blood pressure; MBP, mean blood pressure; PP, pulse pressure; HR, heart rate; MVR, muscular vessel ratio.

POW, percentage of overweight; BMI, body mass index. * *p* < 0.05 vs. underweight, ** *p* < 0.05 vs. -normal, † *p* < 0.05 vs. +normal, ‡ *p* < 0.05 vs. overweight

**Supplementary Table 2b. Cardio-ankle vascular index of participants by body fat percentage classification (Female, *n* = 289)**

| **Body fat percentage** **categories** | **Total** | **Underweight** | **-Normal** | **+Normal** | **Overweight** | **Obese** | ***p* value** |
| --- | --- | --- | --- | --- | --- | --- | --- |
| **Body fat percentage (%)** | 20.0 ± 9.6 | 7.4 ± 3.4 | 14.8 ± 4.6 | 22.9 ± 4.7 | 29.6 ± 3.5 | 39.2 ± 6.2 | < 0.001 |
| ***n*** | 289 | 10 | 152 | 87 | 20 | 20 | - |
| **CAVI** | 4.6 ± 0.6 | 4.8 ± 0.6 | 4.8 ± 0.6 | 4.5 ± 0.7 | 4.4 ± 0.6 | 4.3 ± 0.6 ^**^ | 0.002 |
| **Age, *n* (%)** | 10.7 ± 2.6 | 11.3 ± 2.2 | 10.6 ± 2.6 | 10.7 ± 2.7 | 10.2 ± 2.2 | 11.5 ± 2.3 | 0.514 |
| **SBP (mmHg)** | 116.3 ± 9.9 | 113.0 ± 8.7 | 114.5 ± 9.9 | 117.6 ± 9.2 | 119.7 ± 9.2 | 122.8 ± 10.4 ^**^ | <0.001 |
| **DBP (mmHg)** | 69.0 ± 6.6 | 65.4 ± 4.9 | 68.3 ± 7.2 | 69.8 ± 5.8 | 71.2 ± 6.3 | 70.3 ± 5.7 | 0.071 |
| **MBP (mmHg)** | 84.7 ± 7.0 | 81.3 ± 5.6 | 83.7 ± 7.4 | 85.7 ± 6.3 | 87.3 ± 6.9 | 87.8 ± 6.5 | 0.007 |
| **PP (mmHg)** | 47.3 ± 7.5 | 47.6 ± 6.8 | 46.2 ± 7.5 | 47.8 ± 7.2 | 48.6 ± 5.8 | 52.5 ± 8.3 ^**^ | 0.007 |
| **HR (bpm)** | 79.9 ± 14.0 | 76.5 ± 14.4 | 79.6 ± 13.8 | 79.7 ± 14.1 | 87.0 ± 13.3 | 78.7 ± 14.4 | 0.198 |
| **MVR (%)** | 48.6 ± 0.4 | 48.6 ± 0.4 | 48.5 ± 0.4 | 48.6 ± 0.4 | 48.6 ± 0.4 | 48.7 ± 0.4 | 0.250 |
| **Height (cm)** | 142.5 ± 14.3 | 140.8 ± 13.3 | 141.0 ± 14.7 | 144.2 ± 14.4 | 141.5 ± 13.1 | 149.0 ± 11.0 | 0.118 |
| **Body weight (kg)** | 38.5 ± 12.9 | 29.9 ± 7.1 | 33.7 ± 9.9 | 41.5 ± 11.8 ^*, **^ | 45.0 ± 11.1 ^*, **^ | 59.2 ± 13.8 ^*, **, †, ‡^ | <0.001 |
| **Rohrer index (kg/m^3^)** | 129.1 ± 21.4 | 105.9 ± 7.8 | 117.2 ± 9.2 ^*^ | 135.1 ± 12.4 ^*, **^ | 156.5 ± 10.0 ^*, **, †^ | 177.2 ± 26.1 ^*, **, †, ‡^ | <0.001 |
| **POW (%)** | 1.7 ± 16.9 | -16.0 ± 6.7 | -8.1 ± 6.4 | 6.6 ± 9.1 ^*, **^ | 24.1 ± 7.2 ^*, **, †^ | 40.9 ± 20.0 ^*, **, †, ‡^ | <0.001 |
| **BMI (kg/m^2^)** | 18.4 ± 3.6 | 14.8 ± 1.0 | 16.5 ± 1.9 | 19.5 ± 2.5 ^*, **^ | 22.1 ± 1.8 ^*, **, †^ | 26.4 ± 4.1 ^*, **, †, ‡^ | <0.001 |
| **Body fat mass (kg)** | 9.5 ± 6.7 | 3.8 ± 1.6 | 6.3 ± 3.1 | 10.9 ± 4.7 ^*, **^ | 14.7 ± 5.0 ^*, **, †^ | 25.3 ± 9.0 ^*, **, †, ‡^ | <0.001 |
| **Lean body mass (kg)** | 29.0 ± 7.2 | 26.1 ± 5.6 | 27.4 ± 7.0 | 30.7 ± 7.3 ^**^ | 30.3 ± 6.3 | 33.9 ± 5.4 ^*, **^ | <0.001 |

CAVI, cardio-ankle vascular index; SBP, systolic blood pressure; DBP, diastolic blood pressure; MBP, mean blood pressure; PP, pulse pressure; HR, heart rate; MVR, muscular vessel ratio.

POW, percentage of overweight; BMI, body mass index. * *p* < 0.05 vs. underweight, ** *p* < 0.05 vs. -normal, † *p* < 0.05 vs. +normal, ‡ *p* < 0.05 vs. overweight

**Supplementary Table 3a. Cardio-ankle vascular index of participants by percentage of overweight classification (Male, *n* = 289)**

| **POW categories** | **Total** | **Underweight** | **Normal** | **Mild obesity** | **Moderate obesity** | **Severe obesity** | ***p* value** |
| --- | --- | --- | --- | --- | --- | --- | --- |
| **POW (%)** |  | POW ≤ -20 | -20 < POW < +20 | +20 ≤ POW <+30 | +30 ≤ POW < +50 | +50 ≤ POW |  |
| **POW (%)** | 3.8 ± 18.9 | -23.3 ± 3.8 | -1.6 ± 10.0 | 23.3 ± 2.9 | 37.7 ± 5.9 | 60.8 ± 15.0 | <0.001 |
| ***n*** | 289 | 9 | 238 | 11 | 20 | 11 | - |
| **CAVI** | 4.8 ± 0.7 | 5.3 ± 0.9 | 4.8 ± 0.6 | 4.8 ± 0.5 | 4.5 ± 0.5 ^*^ | 4.2 ± 1.0 ^*, **^ | <0.001 |
| **Age, *n* (%)** | 10.4 ± 2.7 | 11.0 ± 3.0 | 10.3 ± 2.7 | 11.2 ± 1.9 | 10.8 ± 2.0 | 11.8 ± 2.8 | 0.231 |
| **SBP (mmHg)** | 117.6 ± 11.0 | 116.4 ± 10.0 | 116.6 ± 10.7 | 121.6 ± 4.1 | 121.6 ± 12.1 | 128.2 ± 14.5 ^**^ | 0.002 |
| **DBP (mmHg)** | 68.4 ± 6.7 | 66.7 ± 6.1 | 68.1 ± 6.5 | 71.3 ± 7.0 | 69.2 ± 7.4 | 70.6 ± 9.5 | 0.360 |
| **MBP (mmHg)** | 84.8 ± 7.3 | 83.3 ± 7.0 | 84.3 ± 7.0 | 88.1 ± 5.8 | 86.6 ± 7.8 | 89.8 ± 10.4 | 0.036 |
| **PP (mmHg)** | 49.2 ± 8.9 | 49.8 ± 6.3 | 48.5 ± 8.7 | 50.4 ± 4.9 | 52.4 ± 10.6 | 57.6 ± 10.1 ^**^ | 0.006 |
| **HR (bpm)** | 77.8 ± 15.3 | 72.4 ± 7.1 | 76.7 ± 14.7 | 88.5 ± 29.6 | 81.7 ± 11.0 | 86.4 ± 12.1 | 0.015 |
| **MVR (%)** | 48.6 ± 0.4 | 48.8 ± 0.4 | 48.6 ± 0.5 | 48.6 ± 0.1 | 48.7 ± 0.4 | 48.8 ± 0.4 | 0.506 |
| **Height (cm)** | 142.9 ± 17.7 | 152.1 ± 18.1 | 141.7 ± 18.0 | 144.3 ± 15.0 | 146.5 ± 12.0 | 152.2 ± 19.0 | 0.116 |
| **Body weight (kg)** | 39.2 ± 15.8 | 33.6 ± 10.9 | 36.3 ±13.3 | 46.9 ± 14.4 | 54.7 ± 13.0 ^*, **^ | 71.0 ± 23.0 ^*, **, †, ‡^ | <0.001 |
| **Rohrer index (kg/m^3^)** | 129.5 ± 23.5 | 93.2 ± 8.3 | 123.2 ± 13.0 | 152.2 ± 5.2 | 171.7 ± 8.5 | 196.7 ± 22.0 | <0.001 |
| **BMI (kg/m^2^)** | 18.5 ± 4.0 | 14.1 ± 1.6 | 17.4 ± 2.5 ^*^ | 21.9 ± 2.0 ^*, **^ | 25.1 ± 1.9 ^*, **, †^ | 29.8 ± 3.5 ^*, **, †, ‡^ | <0.001 |
| ***BIA n = 272*** | 272 | 10 | 61 | 147 | 23 | 31 | - |
| **Body fat percentage (%)** | 17.5 ± 10.3 | 5.3 ± 2.5 | 14.4 ± 6.2 ^*^ | 27.4 ± 3.0 ^*, **^ | 37.0 ± 3.4 ^*, **, †^ | 43.2 ± 7.7 ^*, **, †, ‡^ | <0.001 |
| **Body fat mass (kg)** | 8.0 ± 7.8 | 1.8 ± 1.1 | 5.7 ± 4.0 | 13.0 ± 4.7 ^*, **^ | 20.3 ± 5.3 ^*, **, †^ | 31.0 ± 12.9 ^*, **, †, ‡^ | <0.001 |
| **Lean body mass (kg)** | 31.6 ± 10.5 | 30.3 ± 9.9 | 30.9 ± 10.4 | 33.9 ± 9,9 | 34.5 ± 8.3 | 40.1 ± 12.8 ^**^ | 0.032 |

POW, percentage of overweight; CAVI, cardio-ankle vascular index; SBP, systolic blood pressure; DBP, diastolic blood pressure; MBP, mean blood pressure; PP, pulse pressure.

HR, heart rate; MVR, muscular vessel ratio; BMI, body mass index; BIA, bioelectrical impedance analysis.

* *p* < 0.05 vs. underweight, ** *p* < 0.05 vs. normal, † *p* < 0.05 vs. mild obesity, ‡ *p* < 0.05 vs. moderate obesity

**Supplementary Table 3b. Cardio-ankle vascular index of participants by percentage of overweight classification (Female, *n* = 301)**

| **POW categories** | **Total** | **Underweight** | **Normal** | **Mild obesity** | **Moderate obesity** | **Severe obesity** | ***p* value** |
| --- | --- | --- | --- | --- | --- | --- | --- |
| **POW (%)** |  | POW ≤ -20 | -20 < POW < +20 | +20 ≤ POW <+30 | +30 ≤ POW < +50 | +50 ≤ POW |  |
| **POW (%)** | 1.4 ± 16.7 | -21.7 ± 1.5 | -2.6 ± 9.5 | 23.8 ± 2.4 | 38.0 ± 6.4 | 64.7 ± 10.0 |  |
| ***n*** | 301 | 11 | 253 | 18 | 13 | 6 | - |
| **CAVI** | 4.6 ± 0.6 | 5.0 ± 0.5 | 4.7 ± 0.6 | 4.4 ± 0.5 ^*^ | 4.4 ± 0.5 | 3.8 ± 0.5 ^*, **^ | <0.001 |
| **Age, *n* (%)** | 10.6 ± 2.6 | 12.3 ± 2.0 | 10.5 ± 2.6 | 11.0 ± 2.6 | 10.5 ± 2.1 | 11.7 ± 2.6 | 0.149 |
| **SBP (mmHg)** | 116.2 ± 9.9 | 113.9 ± 10.4 | 115.6 ± 9.6 | 120.8 ± 10.3 | 117.9 ± 7.3 | 130.2 ± 12.2 ^*, **^ | 0.001 |
| **DBP (mmHg)** | 69.1 ± 6.6 | 64.4 ± 5.4 | 69.1 ± 6.6 | 70.0 ± 6.1 | 69.8 ± 6.9 | 72.3 ± 6.7 | 0.110 |
| **MBP (mmHg)** | 84.8 ± 7.0 | 80.9 ± 6.2 | 84.6 ± 7.0 | 86.9 ± 6.6 | 85.8 ± 6.6 | 91.6 ± 8.0 ^*^ | 0.022 |
| **PP (mmHg)** | 47.2 ± 7.5 | 49.6 ± 8.9 | 46.5 ± 7.2 | 50.8 ± 8.5 | 48.2 ± 5.4 | 57.8 ± 8.4 ^**^ | <0.001 |
| **HR (bpm)** | 79.9 ± 13.8 | 72.7 ± 15.4 | 80.2 ± 13.8 | 79.8 ± 12.3 | 80.2 ± 15.2 | 81.3 ± 14.2 | 0.532 |
| **MVR (%)** | 48.6 ± 0.4 | 48.7 ± 0.3 | 48.6 ± 0.4 | 48.6 ± 0.4 | 48.7 ± 0.4 | 48.6 ± 0.3 | 0.469 |
| **Height (cm)** | 142.2 ± 14.3 | 150.7 ± 8.3 | 141.4 ± 14.6 | 145.2 ± 14.0 | 142.3 ± 12.4 | 151.4 ± 6.9 | 0.097 |
| **Body weight (kg)** | 38.1 ± 12.9 | 34.5 ± 5.2 | 36.1 ± 11.3 | 48.9 ± 13.0 ^*, **^ | 51.0 ± 12.4 ^*, **^ | 72.1 ± 9.9 ^*, **, †, ‡^ | <0.001 |
| **Rohrer index (kg/m^3^)** | 128.7 ± 21.2 | 100.0 ± 3.3 | 123.8 ± 12.4 ^*^ | 156.4 ± 5.8 ^*, **^ | 174.2 ± 10.8 ^*, **, †^ | 206.7 ± 13.7 ^*, **, †, ‡^ | <0.001 |
| **BMI (kg/m^2^)** | 18.3 ± 3.6 | 15.1 ± 0.9 | 17.5 ± 2.5 ^*^ | 22.7 ± 2.1 ^*, **^ | 24.8 ± 2.2 ^*, **^ | 31.3 ± 2.3 ^*, **, †, ‡^ | <0.001 |
| ***BIA n = 289*** | 289 | 11 | 241 | 18 | 13 | 6 | - |
| **Body fat percentage (%)** | 22.4 ± 8.2 | 15.8 ± 2.5 | 20.5 ± 6.1 | 32.2 ± 4.6 ^*, **^ | 36.6 ± 5.5 ^*, **^ | 48.3 ± 2.7 ^*, **, †, ‡^ | <0.001 |
| **Body fat mass (kg)** | 9.5 ± 6.7 | 5.5 ± 1.4 | 8.0 ± 4.5 | 16.1 ± 6.1 ^*, **^ | 19.1 ± 6.9 ^*, **^ | 35.0 ± 6.4 ^*, **, †, ‡^ | <0.001 |
| **Lean body mass (kg)** | 29.0 ± 7.2 | 29.0 ± 4.3 | 28.4 ± 7.2 | 32.8 ± 7.5 | 31.9 ± 6.4 | 37.1 ± 3.7 ^**^ | 0.002 |

POW, percentage of overweight; CAVI, cardio-ankle vascular index; SBP, systolic blood pressure; DBP, diastolic blood pressure; MBP, mean blood pressure; PP, pulse pressure.

HR, heart rate; MVR, muscular vessel ratio; BMI, body mass index; BIA, bioelectrical impedance analysis.

* *p* < 0.05 vs. underweight, ** *p* < 0.05 vs. normal, † *p* < 0.05 vs. mild obesity, ‡ *p* < 0.05 vs. moderate obesity

**Supplementary Table 4a. Cardio-ankle vascular index of participants by body mass index classification (Male, *n* = 289)**

| **Body mass index categories** | **Total** | **Underweight** | **Normal weight** | **Overweight to obesity** | ***p* value** |
| --- | --- | --- | --- | --- | --- |
| **Body mass index (kg/m^2^)** |  | Body mass index ˂ 18.5 | 18.5 ≤ Body mass index ˂ 25 | 25 ≤ Body mass index |  |
| **Body mass index (kg/m^2^)** | 18.5 ± 4.0 | 16.1 ± 1.4 | 21.3 ± 1.7 | 28.5 ± 3.1 | <0.001 |
| ***n*** | 289 | 183 | 86 | 20 | - |
| **CAVI** | 4.8 ± 0.7 | 4.8 ± 0.7 | 4.7 ± 0.6 | 4.3 ± 0.9 ^*, **^ | 0.003 |
| **Age, *n* (%)** | 10.4 ± 2.7 | 9.6 ± 2.7 | 11.7 ± 2.0 ^*^ | 12.4 ± 2.1 ^*^ | <0.001 |
| **SBP (mmHg)** | 117.6 ± 11.0 | 114.3 ± 10.1 | 121.6 ± 9.2 ^*^ | 130.0 ± 11.9 ^*, **^ | <0.001 |
| **DBP (mmHg)** | 68.4 ± 6.7 | 67.9 ± 6.7 | 68.7 ± 5.9 | 70.6 ± 9.4 | 0.203 |
| **MBP (mmHg)** | 84.8 ± 7.3 | 83.4 ± 7.2 | 86.4 ± 6.0 ^*^ | 90.4 ± 9.6 ^*^ | <0.001 |
| **PP (mmHg)** | 49.2 ± 8.9 | 46.4 ± 7.7 | 52.9 ± 8.6 ^*^ | 59.4 ± 8.2 ^*, **^ | <0.001 |
| **HR (bpm)** | 77.8 ± 15.3 | 78.5 ± 14.3 | 73.9 ± 14.1 | 88.1 ± 22.7 ^*, **^ | <0.001 |
| **MVR (%)** | 48.6 ± 0.4 | 48.5 ± 0.5 | 48.8 ± 0.3 ^*^ | 48.9 ± 0.3 ^*^ | <0.001 |
| **Height (cm)** | 142.9 ± 17.7 | 136.8 ± 17.1 | 152.5 ± 13.6 ^*^ | 156.7 ± 13.4 ^*^ | <0.001 |
| **Body weight (kg)** | 39.2 ± 15.8 | 30.8 ± 9.5 | 49.8 ± 9.7 ^*^ | 70.8 ± 16.6 ^*, **^ | <0.001 |
| **Rohrer index (kg/m^3^)** | 129.5 ± 23.5 | 118.5 ± 13.4 | 140.6 ± 18.1 ^*^ | 182.6 ± 22.8 ^*, **^ | <0.001 |
| **POW (%)** | 3.8 ± 18.9 | -6.3 ± 8.7 | 14.6 ± 12.5 ^*^ | 49.6 ± 17.1 ^*, **^ | <0.001 |
| ***BIA n = 272*** | 272 | 169 | 83 | 20 | - |
| **Body fat percentage (%)** | 17.5 ± 10.3 | 11.4 ± 4.3 | 24.3 ± 7.3 ^*^ | 40.1 ± 7.3 ^*, **^ | <0.001 |
| **Body fat mass (kg)** | 8.0 ± 7.8 | 3.6 ± 1.7 | 11.9 ± 3.8 ^*^ | 28.5 ± 9.8 ^*, **^ | <0.001 |
| **Lean body mass (kg)** | 31.6 ± 10.5 | 27.3 ± 8.6 | 37.9 ± 8.9 ^*^ | 42.2 ± 9.9 ^*^ | <0.001 |

CAVI, cardio-ankle vascular index; SBP, systolic blood pressure; DBP, diastolic blood pressure; MBP, mean blood pressure; PP, pulse pressure; HR, heart rate.

MVR, muscular vessel ratio; POW, percentage of overweight; BIA, bioelectrical impedance analysis.

* *p* < 0.05 vs. underweight, ** *p* < 0.05 vs. normal weight

**Supplementary Table 4b. Cardio-ankle vascular index of participants by body mass index classification (Female, *n* = 301)**

| **Body mass index categories** | **Total** | **Underweight** | **Normal weight** | **Overweight to obesity** | ***p* value** |
| --- | --- | --- | --- | --- | --- |
| **Body mass index (kg/m^2^)** |  | Body mass index ˂ 18.5 | 18.5 ≤ Body mass index ˂ 25 | 25 ≤ Body mass index |  |
| **Body mass index (kg/m^2^)** | 18.3 ± 3.6 | 15.9 ± 1.4 | 20.7 ± 1.7 | 28.1 ± 3.0 | <0.001 |
| ***n*** | 301 | 177 | 108 | 16 |  |
| **CAVI** | 4.6 ± 0.6 | 4.7 ± 0.6 | 4.7 ± 0.6 | 4.2 ± 0.6 ^*, **^ | 0.016 |
| **Age, *n* (%)** | 10.6 ± 2.6 | 9.5 ± 2.3 | 12.1 ± 2.2 ^*^ | 12.5 ± 1.8 ^*^ | <0.001 |
| **SBP (mmHg)** | 116.2 ± 9.9 | 113.8 ± 9.0 | 119.0 ± 9.7 ^*^ | 124.4 ± 10.6 ^*^ | <0.001 |
| **DBP (mmHg)** | 69.1 ± 6.6 | 68.4 ± 6.8 | 69.8 ± 6.2 | 70.7 ± 7.3 | 0.130 |
| **MBP (mmHg)** | 84.8 ± 7.0 | 83.5 ± 6.8 | 86.2 ± 6.8 ^*^ | 88.6 ± 7.8 ^*^ | <0.001 |
| **PP (mmHg)** | 47.2 ± 7.5 | 45.3 ± 7.1 | 49.2 ± 7.0 ^*^ | 53.8 ± 7.5 ^*, **^ | <0.001 |
| **HR (bpm)** | 79.9 ± 13.8 | 81.7 ± 13.2 | 77.7 ± 14.6 | 74.6 ± 13.0 | 0.017 |
| **MVR (%)** | 48.6 ± 0.4 | 48.4 ± 0.4 | 48.7 ± 0.3 ^*^ | 48.9 ± 0.3 ^*^ | <0.001 |
| **Height (cm)** | 142.2 ± 14.3 | 136.2 ± 13.8 | 150.4 ± 10.4 ^*^ | 153.5 ± 7.2 ^*^ | <0.001 |
| **Body weight (kg)** | 38.1 ± 12.9 | 30.1 ± 7.7 | 47.1 ± 7.3 ^*^ | 66.1 ± 8.4 ^*, **^ | <0.001 |
| **Rohrer index (kg/m^3^)** | 128.7 ± 21.2 | 117.8 ± 11.2 | 138.5 ± 15.4 ^*^ | 183.2 ± 23.0 ^*, **^ | <0.001 |
| **POW (%)** | 1.4 ± 16.7 | -7.3 ± 8.0 | 9.2 ± 12.7 ^*^ | 45.1 ± 18.1 ^*, **^ | <0.001 |
| ***BIA n = 289*** | 289 | 168 | 105 | 16 |  |
| **Body fat percentage (%)** | 22.4 ± 8.2 | 17.1 ± 3.7 | 27.8 ± 4.8 ^*^ | 42.3 ± 6.4 ^*, **^ | <0.001 |
| **Body fat mass (kg)** | 9.5 ± 6.7 | 5.4 ± 2.3 | 13.2 ± 3.6 ^*^ | 28.3 ± 7.3 ^*, **^ | <0.001 |
| **Lean body mass (kg)** | 29.0 ± 7.2 | 25.1 ± 5.9 | 33.9 ± 5.0 ^*^ | 37.8 ± 3.5 ^*, **^ | <0.001 |

CAVI, cardio-ankle vascular index; SBP, systolic blood pressure; DBP, diastolic blood pressure; MBP, mean blood pressure; PP, pulse pressure; HR, heart rate.

MVR, muscular vessel ratio; POW, percentage of overweight; BIA, bioelectrical impedance analysis.

* *p* < 0.05 vs. underweight, ** *p* < 0.05 vs. normal weight
